# Diabetes is associated with higher mortality and severity in hospitalized patients with COVID-19

**DOI:** 10.17179/excli2021-3403

**Published:** 2021-02-22

**Authors:** Fatemeh Moghaddam Tabrizi, Yousef Rasmi, Elyas Hosseinzadeh, Sakineh Rezaei, Mohadeseh Balvardi, Mohammad Reza Kouchari, Ghasem Ebrahimi

**Affiliations:** 1 Reproductive Health Research Center, Urmia University of Medical Sciences, Urmia, Iran; 2 Khoy University of Medical Sciences, Khoy, Iran; 3 Cellular and Molecular Research Center, Urmia University of Medical Sciences, Urmia, Iran; 4 Department of Biochemistry, Faculty of Medicine, Urmia University of Medical Sciences, Urmia, Iran; 5 Department of Laboratory Sciences, Sirjan School of Medical Sciences, Sirjan, Iran; 6 Ayatoollah Khoyi Hospital, Khoy University of Medical Sciences, Khoy, Iran; 7 Instructor of Biostatistics, Sirjan School of Medical Sciences, Sirjan, Iran; 8 Department of Biochemistry and Clinical Laboratories, Faculty of Medical Sciences, Tabriz University of Medical Sciences, Tabriz, Iran

**Keywords:** diabetes, comorbidity, inflammation, mortality, COVID-19

## Abstract

As a novel cause of pneumonia, coronavirus disease 2019 (COVID-19) has rapidly progressed worldwide. Previous studies have indicated COVID-19 patients with diabetes show higher mortality rates and more severe COVID-19 infection with an increased requirement for intensive care and hospital length of stay (LOS) compared to non-diabetic patients. The present study aimed to investigate the association of diabetes and COVID-19 outcome with severity of disease in hospitalized patients. The present case-control study included 268 patients diagnosed with COVID-19 who were hospitalized in Ayatollah Khoyi Hospital, Khoy, Iran. Diabetes was identified based on medical history and/or criteria of published documents. Out of 268 patients (median age of 59 years; 53.4 % male), 127 patients had diabetes (47 %). Diabetic patients had remarkably higher mortality rates (adjusted odds ratio, aOR: 3.36; confidence interval, CI: 1.17-9.66), requirement for invasive mechanical ventilation (IMV) (aOR: 4.59; CI: 1.38-15.25), and LOS (aOR: 1.13; CI: 1.06-1.24) compared to patients without diabetes. Inflammatory biomarkers including C-reactive protein (CRP), lactate dehydrogenase (LDH), and erythrocyte sedimentation rate (ESR) were increased in patients with diabetes compared to non-diabetic patients (P < 0.05 for all the comparisons). In hospitalized patients with COVID-19, diabetes was correlated with increased disease severity and mortality.

## Introduction

Severe acute respiratory syndrome coronavirus-2 (SARS-CoV-2) is a novel and highly contagious coronavirus, which has caused an unprecedented outbreak of coronavirus disease 2019 (COVID-19) and mostly attacks multiorgan systems, including the lower respiratory tract (Wu and McGoogan, 2020[30]; Zhu et al., 2020[41]). Patients with severe COVID-19 showed evidence of various organ involvement, acute respiratory distress syndrome (ARDS), and systemic complications (Guan et al., 2020[13]). 

Accumulating evidence has demonstrated that elderly patients with pre-existing diseases, such as hypertension, diabetes, and cardiovascular disease (CVD), are infected with a more severe form of COVID-19 and therefore are at higher risk of fatality (Wang et al., 2020[29]; Zhang et al., 2020[38]; Zhou et al., 2020[39]). Of these diseases, diabetes has previously been recognized as a common comorbidity present in COVID-19 patients (Zhu et al., 2020[40]). The co-occurrence of this viral pandemic with the growing incidence of diabetes worldwide has created a serious challenge for human kind (Zhou et al., 2020[39]). A one-month follow-up study showed that good glycemic control in diabetic patients with COVID-19 is associated with better survival in the patients (Zhu et al., 2020[40]). However, wide gaps remain in our knowledge about the association of diabetes with a poor prognosis of COVID-19 and this relation has yet to be thoroughly investigated. Therefore, more studies are needed to confirm poor prognosis in diabetic patients with severe COVID-19 infection and develop effective treatment approaches.

Diabetes is a common systemic disease with serious multi-systemic complications. It generally increases the susceptibility to infection, and subsequently leads to poorer prognosis in diabetic patients infected with viral diseases compared to non-diabetic patients (Huang et al., 2020[18]; Kumar Nathella and Babu, 2017[21]; Xu et al., 2019[32]; Zhu et al., 2020[40]). Past viral infections, such as previous coronaviral epidemics, are the most obvious examples indicating the association of diabetes with a high rate of morbidity and mortality. For example, pre-existing diabetes has been shown to be a critical risk factor associated with a higher death rate in patients with SARS (Booth et al., 2003[3]; Yang et al., 2006[35]). In the Middle East, in the cases of respiratory syndrome coronavirus (MERS-CoV) infection, diabetes was also the most common comorbidity with a higher mortality rate and severity of illness (Alqahtani et al., 2019[2]). Regarding the COVID-19 pandemic, there is a growing body of evidence supporting the connection between diabetes and COVID-19-related death, and a large proportion of intensive care unit (ICU)-admitted COVID-19 patients have been reported to have pre-existing diabetes (Deng and Peng, 2020[7]; Wang et al., 2020[29]; Zhang et al., 2020[37]; Zhou et al., 2020[39]).

Given the fact that hyperglycemia deteriorates inflammation and viremia, which lead to severe clinical complications (Bornstein et al., 2020[4]; Forbes et al., 2018[10]), it could be postulated that hyperglycemia is involved in the pathogenesis of COVID-19 as well. Thus, it seems that glycemic fluctuation is the most common complication associated with serious morbidity and mortality in patients infected by SARS-CoV-2. Therefore, the present study aimed to assess the association between diabetes and clinical endpoints in SARS-CoV-2-infected patients. 

## Methods

### Participants and study design

In the present case-control study, a total of 268 COVID-19 cases (confirmed by RT-PCR, CT-scan, and laboratory data), hospitalized in Ayatollah Khoyi Hospital in Khoy, West Azerbaijan, Iran between March 20^th^ and July 21^st^, 2020, were included. The study was reviewed by the Institutional Ethics Committee of the Urmia University of Medical Sciences, Urmia, Iran, and a waiver of consent was allowed due to the retrospective nature of the study. Data from 320 hospitalized patients infected with SARS-CoV-2 were analyzed for the study. Subjects with the ages between 18 and 75 years old who did not have an acute lethal organ injury, end-stage chronic organ failure, and malignancy and were not pregnant were selected for the study. Based on the laboratory data, clinical findings, and/or medical history on admission, 268 patients out of 320 initial hospitalized patients were qualified for the study. The recruited patients were divided into two groups including diabetic (n = 127) and non-diabetic (n = 141) patients for further evaluation.

### Data collection and definition 

Analyses of the medical records of the participants were carried out by physicians, data scientists, and statisticians. After assigning a code to each participant using a coding system, the basic information, epidemiological records, and laboratory findings, as well as data on clinical manifestations, CT scan results, treatments, and in-hospital outcomes were collected. Patients with severe conditions were defined based on the respiratory rate > 30 breaths/min, or oxygen saturation (SpO_2_) ≤ 93 % on room air. Diabetes status was defined according to the patient's medical history and criteria such as fasting blood glucose ≥ 126 mg/dL or casual plasma glucose ≥ 200 mg/dL (Burtis et al., 2012[6]; Zhu et al., 2020[40]). Hypertension was indicated as systolic blood pressure equal to or above 140 mmHg and diastolic blood pressure equal to or above 90 mmHg or defined based on medical documents from a previously published study (Zhu et al., 2020[40]). Obesity was determined as body mass index (BMI) ≥ 30. Inflammatory markers with appropriate cutoff values, i.e., C-reactive protein (CRP) ˃ 41.2 mg/L and lactate dehydrogenase (LDH) ˃ 365 units/L, which have previously been described to be associated with mortality in patients with COVID-19, were selected from published papers (Xie et al., 2020[31]; Yan et al., 2020[33]). Leucocyte or white blood cell (WBC) count and the neutrophil-to-lymphocyte ratio (NLR), as biomarkers for evaluating the immune system imbalance, were calculated based on a previously published work (Yang et al., 2020[34]). 

### Statistical analysis

Categorical and continuous variables were presented as the median and interquartile range (IQR) and numbers (percent), respectively. Categorical variables were analyzed using chi-square or Fisher's exact test. Comparison of continuous variables between diabetic and non-diabetic patients was carried out using the independent sample t-test or the Mann-Whitney U test. The risk for composite endpoints and the corresponding odds ratios (OR) were analyzed using a multivariable logistic regression model. Statistical analysis was performed using SPSS software version 19 (IBM statistic, New York, NY, USA). The multivariable logistic regression model was developed using SAS (Statistical Analysis System) version 9.2. The significance level was set at 0.05.

## Results

### Clinical characteristics 

Comparison of clinical features of 268 confirmed COVID-19 patients, including 127 cases with diabetes (68 males, 53.5 %) and 141 cases without diabetes (75 males, 53.2 %) is presented in Table 1(Tab. 1). The median ages of diabetic and non-diabetic patients were 64 (55-72) and 49 (39-62) years old, respectively (P < 0.001). Moreover, the median BMI was 30.1 (27.6-32.9) and 27.3 (24.2-29) in diabetic and non-diabetic patients, respectively (P < 0.001). Despite the similar respiratory rate between the two groups, a modest increase in systolic blood pressure was observed in the diabetic group (130 mmHg [110-143] vs. 120 mmHg [110-131]). Cough was the most common manifestation (67.2 %), followed by fatigue (54.1 %), dyspnea (53.7 %), and fever (49.3 %) among all patients. The incidence rates of cough and anosmia were significantly higher in diabetic patients (73.2 % and 15.6 %, respectively; P = 0.045) compared to the non-diabetic group (61.7 % and 6.3 %, respectively; P = 0.016). There was a significantly higher prevalence of comorbidities such as hypertension (59.9 % vs. 25.0 %), coronary heart disease (CHD) (38.9 % vs. 20.0 %), obesity (50.8 % vs. 16.2 %), chronic kidney disease (CKD) (7.1 % vs. 1.4 %), and brain disease (5.0 % vs. 0.8 %) in patients with diabetes compared to the patients without diabetes. Notably, 55.3 % of the non-diabetic patients with COVID-19 did not have any comorbidity, while 11.8 % of the cases with diabetes and COVID-19 had no other comorbidities. Extensive lung injury in chest CT scans, which was indicated by bilateral lung lesions, was higher in the diabetic group than the non-diabetic group with the incidence rates of 93.7 % and 84.3 %, respectively (Table 1(Tab. 1)).

### Laboratory findings

Understandably, laboratory findings showed significantly higher glucose levels in diabetic patients compared to the non-diabetic group (9.4 mmol/L [7.5-11.9] vs. 5.4 mmol/L [4.9-6.1], respectively). Moreover, SpO_2 _rates lower than 95 % were found to be more prevalent in the diabetic patients in comparison to the non-diabetic group on admission (80.3 % vs. 65.9 %; P = 0.047). However, elevations in serum parameters, which indicated decreased kidney function (creatinine) and the hypercoagulable state (D-dimer), were not significantly different in the two groups. Our findings indicated that the inflammatory status was significantly elevated in patients with diabetes in comparison with the non-diabetic patients. In this regard, remarkably higher levels of LDH (509 [437.5-665] vs. 465 [362-557]), CRP (95.8 vs. 88.7) and erythrocyte sedimentation rate (ESR) (46 [30-61] vs. 36.5 [23-50]) were found in patients with diabetes as compared to the non-diabetic group (P = 0.019 and P = 0.005, respectively). Similarly, leukocytosis (29.6 % vs. 13.9 %), lymphopenia (44 % vs. 23 %), neutrophilia (39.2 % vs. 16.3 %) and NLR (4.2 [2.7-6.5] vs. 2.6 [1.9-3.2]) were significantly higher in patients with diabetes relative to the non-diabetic patients (Table 1(Tab. 1)).

### Treatments

As shown in Table 2(Tab. 2), diabetic patients with COVID-19 required a higher amount of anti-hypertensive drugs (42.5 % vs. 19.1 %), immunoglobin (12.6 % vs. 2.8 %), antibiotics (83.5 % vs. 51.8 %), and lipid-lowering medications (30 % vs. 13.5 %). Similarly, the frequency of the need for invasive mechanical ventilation (IMV) (18.1 % vs. 2.8 %) was also significantly higher in patients with diabetes compared to non-diabetic patients (P ˂ 0.001).

### Disease severity and in-hospital mortality 

During the hospitalization period, various parameters were analyzed for each patient in the study. Our collected data indicated that, compared to the non-diabetic group, patients with diabetes had higher NLR as well as higher levels of CRP, ESR, LDH, and blood glucose. The findings of this study indicated the unequivocal association of diabetes mellitus with elevated rates of in-hospital death (17.3 % vs. 5.7 % ), higher proportion of patients undergoing IMV (18.1 % vs. 2.8 % ), and higher median levels of the length of the healing process, indicated by the hospital length of stay (LOS) (6 [CI: 4-11] vs. 5 [CI: 4-7]). After adjusting for age and gender, it was shown that diabetes was associated with 3.36-fold increased risk of in-hospital death (CI: 1.17-9.66), 4.59-fold increased odds of IMV requirement (1.38-15.25), and 1.14-fold increased odds of LOS (CI: 1.06-1.24) in patients with COVID-19 in comparison with non-diabetic patients (Table 3(Tab. 3)).

See also the Supplementary data.

## Discussion

The rapid spread of COVID-19 throughout the world and worse outcomes in COVID-19 patients with pre-existing diabetes have raised numerous questions about the contribution of diabetes to this global crisis. It is well established that patients with diabetes have a state of metabolic inflammation, which compromises the ability of the immune system to tackle the infection (Yang et al., 2020[34]; Ying et al., 2014[36]). The findings of the present study revealed a remarkable association between diabetes and worse conditions in patients with COVID-19 infection. It was found that diabetic patients infected with SARS-CoV-2 had an increased immune system imbalance and higher levels of inflammatory biomarkers compared to non-diabetic patients.

Diabetes is considered to be one of the underlying causes of the elevation of leukocyte count, NLR, and infection-associated markers, such as LDH, CRP, and ESR, which cause chronic low-grade inflammation. Of these biomarkers, NLR is considered to be a dynamic parameter, demonstrating the balance of the complementary but paradoxical innate and adaptive immune systems. Several studies have indicated the paradoxical influence of hyperglycemia on lymphocyte and neutrophil counts in diabetic patients, explained by higher apoptosis and oxidative**-**mediated DNA damage in peripheral blood lymphocytes and lower apoptosis in neutrophils (Adaikalakoteswari et al., 2007[1]; Otton et al., 2004[24]). These conditions proceed to impair neutrophil clearance and prolong inflammation (Hanses et al., 2011[15]). On the other hand, both diabetes and virus-induced inflammatory elements, produced by endothelial and lymphocyte cells, cause neutrophil augmentation which can exacerbate the patients' condition. Lymphocytes have been suggested to be the first-line defense of the human immune system against infections triggered by viruses (Rabinowich et al., 1987[25]); however, systematic inflammation created by pre-existing diabetes, causes a significant reduction in the lymphocyte count and a remarkable elevation in the levels of its suppressors (Menges et al., 1999[22]). Therefore, it is reasonable to say that diabetic patients infected with SARS-CoV-2 face severe inflammatory conditions and poor prognosis in advance. In the present study, the obtained data showed that the lymphocyte count in patients with diabetes decreased more significantly than non-diabetic patients, while the neutrophil count increased significantly in diabetic patients in comparison to the non-diabetic ones. Similar results were observed for inflammatory biomarkers including CRP, ESR, and LDH. These results suggest diabetes as a predictor of poor outcome in COVID-19 patients.

As explained earlier, diabetes mellitus has been demonstrated as one of the pivotal factors causing morbidity worldwide. The mechanisms behind this may include higher susceptibility to some infectious diseases (Joshi et al., 1999[19]; Muller et al., 2005[23]; Shah and Hux, 2003[28]), probably through dysregulating the immune system (Hodgson et al., 2015[16]). Given this finding, patients with diabetes have been recommended for pneumococcal and annual influenza vaccinations in the past years (Gupta et al., 2020[14]). In addition, obesity, hypertension, CVDs, and chronic kidney disease are common in patients with diabetes. These comorbidities can stimulate excessive inflammation by predisposing the patients to immune response suppression, infection with diverse viral quasispecies, respiratory tract mechanical dysfunction, sodium-fluid retention, negative effects on glucose metabolism, and insulin resistance, resulting in worse outcomes (Dixon and Peters, 2018[8]; Ferrannini and Cushman, 2012[9]; Gao et al., 2020[12]; Honce et al., 2020[17]; Rizos and Elisaf, 2014[26]; Saltiel and Olefsky, 2017[27]). In this respect, to prevent the deleterious effects of these diabetes-associated comorbidities, more intensive measures such as medicating with different drug categories, including anti-diabetic, anti-hypertensive, lipid-lowering, antiviral, and antibacterial drugs, are necessary for diabetic patients infected with COVID-19. Correspondingly, our data showed that patients with diabetes needed more intensive integrated treatments than the non-diabetic subjects.

Research has proposed that angiotensin-converting enzyme 2 (ACE2) is overexpressed in various tissues of patients taking anti-hypertensive and anti-diabetic medication, which accelerates the SARS-CoV-2 binding, cell infection, and subsequently, SARS-CoV-2-mediated ACE2 reduction (Bornstein et al., 2020[5]). To our knowledge, ACE2 has a key regulatory effect on the renin-angiotensin system in different tissues by degrading angiotensin II into angiotensin 1-7, which in turn decreases inflammation. Considering these facts, the impairment of the protective ACE2 receptor pathway by SARS-COV-2 increases deleterious angiotensin II activity, leading to the induction of pro-inflammatory responses and an increase in blood pressure (Bornstein et al., 2020[4][5]), which result in a higher risk of adverse complications in patients with diabetes and COVID-19.

We also analyzed disease severity in diabetic and non-diabetic patients. The findings of this study indicated the unequivocal association of diabetes mellitus with a more than three-fold increase in the risk of in-hospital mortality and an almost five-fold increase in the odds of IMV requirement in diabetic patients with COVID-19 compared to the non-diabetic ones, after adjusting for age and gender. These results revealed that diabetes is a potential predictor of the worse outcome of COVID-19, which is consistent with the results of the research by the Huang group (Zhu et al., 2020[40]). Adjusting for comorbidities, including obesity, CHD, hypertension, and chronic kidney disease, which are closely related to diabetes, was not entirely appropriate, as these diseases frequently co-exist with diabetes. The findings of this study indicated a clear relationship between diabetes mellitus and higher requirement for ICU admission and IMV, as well as the lengthening of the healing process.

There is an urgent need to fully explore the underlying mechanisms by which diabetes promotes COVID-19 severity. An earlier study carried out by Fuso et al. demonstrated the adverse effects of diabetes on respiratory function abnormalities (Fuso et al., 2019[11]), which delineates a tendency towards worse outcomes in diabetic patients infected with SARS-CoV-2. Moreover, immune deficiency is prevalent in individuals with all types of diabetes and places them at greater risk for morbidity. Nonetheless, it is plausible that immune dysregulation is responsible for increased COVID-19 severity in patients with diabetes, since we observed higher NLR and elevated levels of serum CRP, ESR, and LDH in diabetic patients infected with SARS-CoV-2 in the present study. These results are consistent with the findings of Kulcsar et al. which explained the connection between diabetes and dysregulated immune response observed in other coronavirus infection-triggered pneumonia (Kulcsar et al., 2019[20]). Moreover, our observations are consistent with those of Zhu et al. (2020[40]) and Yan et al. (2020[33]), which suggested the contribution of diabetes to higher mortality, hospital LOS, admission to ICU, and necessity of intensive medication in diabetic patients compared to non-diabetic patients with COVID-19. 

## Conclusion

Diabetes, as an important risk factor, is associated with deleterious comorbidities, inflammation, higher mortality, elevated IMV requirement, and longer hospital stay in patients with COVID-19 compared to non-diabetic patients with COVID-19. This situation enforces more intensive integrated treatment for patients with diabetes which may jeopardize the patients' life by worsening the progression of COVID-19.

## Notes

Elyas Hosseinzadeh and Ghasem Ebrahimi (Department of Biochemistry and Clinical Laboratories, Faculty of Medical Sciences, Tabriz University of Medical Sciences, Tabriz, Iran; Tel: +98936 273 3850, E-mail: gheb67@gmail.com, ebrahimigh@tbzmed.ac.ir) equally contributed as corresponding authors.

## Declaration of competing interest

The authors declare that they have no known competing financial interests or personal relationships that could have appeared to influence the work reported in this paper.

## Acknowledgement

We would like to express our sincere gratitude to Hamed Akbari for his great contribution to this research. This study was supported by the Khoy University of Medical Sciences.

## Authors’ contributions

FMT and YR contributed to the material preparation, study conception and design, and data collection and interpretation. EH and GE contributed to the study conception and design, writing the original draft and data interpretation. SR contributed to data collection. The data analysis was performed by MB. MRK contributed to data collection and interpretation. All authors read and approved the final manuscript.

## Supplementary Material

Supplementary data

## Figures and Tables

**Table 1:**
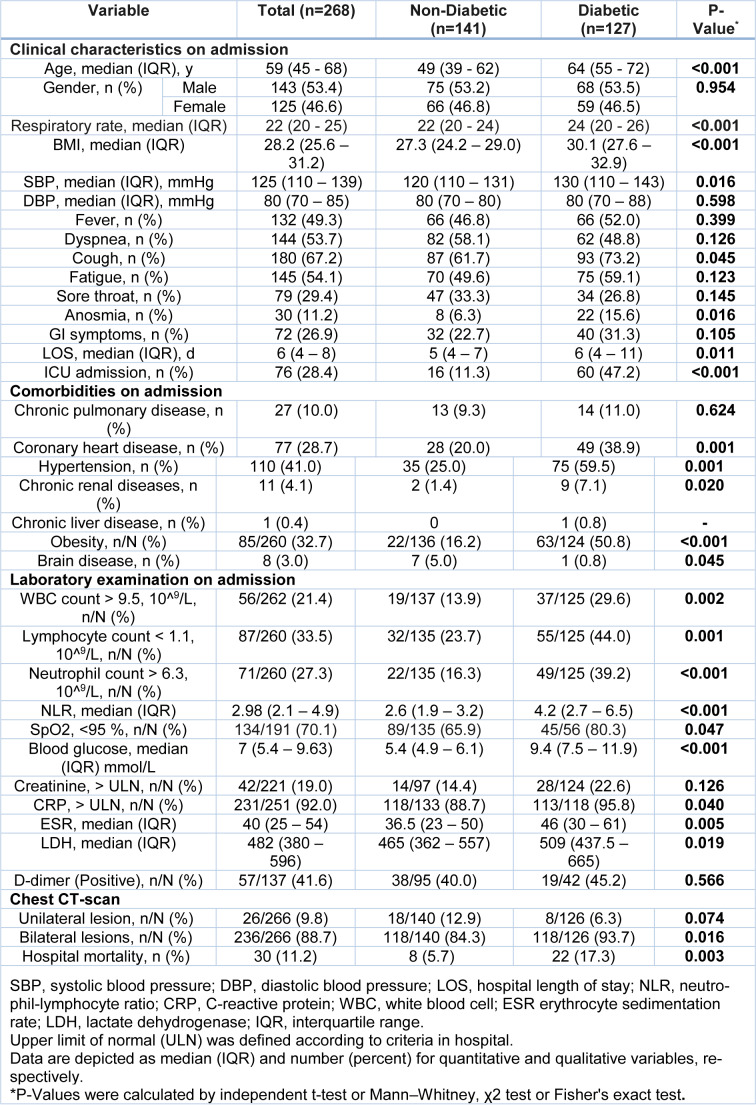
Comparison of clinical characteristics between diabetic and non-diabetic patients with COVID-19

**Table 2:**
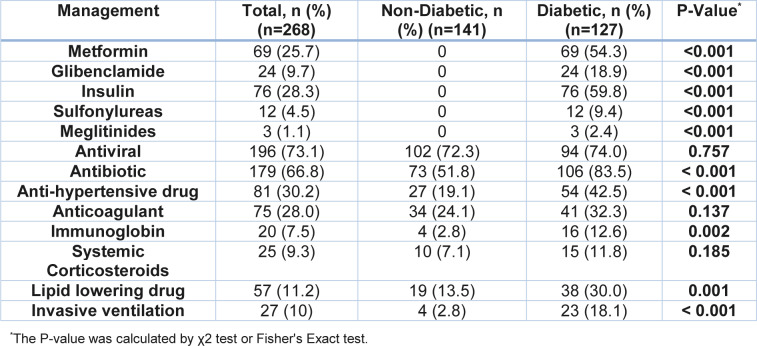
In-hospital management of SARS-CoV-2 infected patients with or without diabetes

**Table 3:**
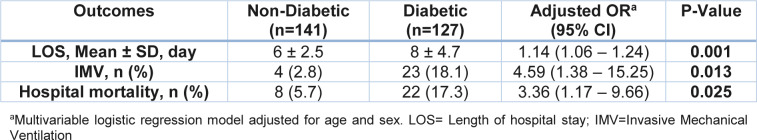
Multivariable analyses, patients with diabetes vs. patients without diabetes
