# Supplementary material for: Diabetes is associated with higher mortality and severity in hospitalized patients with COVID-19
Source: EXCLI J. 2021 Feb 22;20:444–53. doi: 10.17179/excli2021-3403 (PMC7975582; doi:10.17179/excli2021-3403)
Supplement: Supplementary data [file EXCLI-20-444-s-001.pdf]

EXCLI Journal 2021;20:444-453 – ISSN 1611-2156
Received: January 16, 2021, accepted: February 16, 2021, published: February 22, 2021

Supplementary data to:

Original article:

DIABETES IS ASSOCIATED WITH INCREASED MORTALITY AND DISEASE SEVERITY IN HOSPITALIZED PATIENTS WITH COVID-19

Fatemeh Moghaddam Tabrizi[a, b], Yousef Rasmi[c, d], Elyas Hosseinzadeh[e]*, Sakineh Rezaei[f], Mohadeseh Balvardi[g], Mohammad Reza Kouchari[f], Ghasem Ebrahimi[h]*

[a] Reproductive Health Research Center, Urmia University of Medical Sciences, Urmia, Iran
[b] Khoy University of Medical Sciences, Khoy, Iran
[c] Cellular and Molecular Research Center, Urmia University of Medical Sciences, Urmia, Iran
[d] Department of Biochemistry, Faculty of Medicine, Urmia University of Medical Sciences, Urmia, Iran
[e] Department of Laboratory Sciences, Sirjan School of Medical Sciences, Sirjan, Iran
[f] Ayatoollah Khoyi Hospital, Khoy University of Medical Sciences, Khoy, Iran
[g] Instructor of Biostatistics, Sirjan School of Medical Sciences, Sirjan, Iran
[h] Department of Biochemistry and Clinical Laboratories, Faculty of Medical Sciences, Tabriz University of Medical Sciences, Tabriz, Iran

* **Corresponding authors:** Elyas Hosseinzadeh, Department of Laboratory Sciences, Sirjan School of Medical Sciences, Sirjan, Iran. Tel & Fax: +983442234513; Postal cod: 7816883333; E-mail: elyas.hosseinzadeh@gmail.com;
Ghasem Ebrahimi, Department of Biochemistry and Clinical Laboratories, Faculty of Medical Sciences, Tabriz University of Medical Sciences, Tabriz, Iran. Tel: +98936 273 3850; E-mail: gheb67@gmail.com, ebrahimigh@tbzmed.ac.ir.

http://dx.doi.org/10.17179/excli2021-3403

This is an Open Access article distributed under the terms of the Creative Commons Attribution License (http://creativecommons.org/licenses/by/4.0/).

EXCLI Journal 2021;20:444-453 – ISSN 1611-2156
Received: January 16, 2021, accepted: February 16, 2021, published: February 22, 2021

Supplementary Table 1: Raw data of clinical characteristics of diabetic and non-diabetic patients with COVID-19 which is shown in Table 1 of the manuscript.

ID	Group 1: Control 2: Case	Age	Gender 1: M 2: F	Respiratory rate	BMI	SBP	DBP	Fever 1: No 2: Yes	Dyspnea 1: No 2: Yes	Cough 1: No 2: Yes	Fatigue 1: No 2: Yes	Sore throat 1: No 2: Yes	Anosmia 1: No 2: Yes	GI symptoms 1: No 2: Yes	LOS	ICU admis-sion 1: General unit 2: ICU
1	1	80	2	20	miss	120	80	1	1	2	1	2	2	1	5	1
2	1	77	2	miss	miss	miss	miss	1	2	2	1	1	1	1	3	1
3	1	66	2	26	27.68	165	100	2	2	2	2	1	1	1	5	2
4	1	75	1	20	24.39	80	50	1	2	2	1	1	1	1	3	1
5	1	34	1	26	27.77	110	75	1	2	1	2	1	1	1	3	1
6	1	39	1	miss	miss	miss	miss	1	2	2	1	1	1	2	7	2
7	1	40	1	35	27.76	140	78	1	2	2	1	1	1	1	7	2
8	1	36	1	24	24.22	120	80	1	2	2	2	1	1	1	8	1
9	1	36	1	24	miss	120	80	1	1	2	1	1	1	2	5	2
10	1	47	1	21	30.86	110	70	2	2	1	1	1	2	1	1	2
11	1	27	1	miss	26.73	miss	miss	2	1	2	1	1	1	1	5	1
12	1	miss	1	18	23.88	100	60	2	1	2	1	1	1	1	4	1
13	1	55	1	18	25.56	110	80	1	1	1	1	1	1	1	4	1
14	1	20	2	22	23.56	104	70	1	1	1	2	1	2	1	4	1
15	1	74	1	20	22.49	120	90	2	2	2	2	1	2	1	4	2
16	1	64	1	18	31.25	110	70	2	2	1	1	1	1	1	6	1
17	1	40	1	20	23.77	110	70	1	1	1	1	1	1	2	9	1
18	1	26	1	20	25.00	120	80	1	2	1	2	1	1	1	5	1
19	1	64	2	22	23.44	110	70	2	2	1	2	1	1	1	6	1
20	1	26	2	22	25.81	110	70	1	1	2	2	1	1	2	8	1
21	1	60	1	20	31.25	130	80	2	1	1	1	1	1	1	6	1
22	1	77	2	24	27.34	130	90	2	1	2	2	2	1	2	4	1
23	1	67	2	25	32.85	110	70	2	2	2	2	2	1	2	5	2
24	1	40	1	19	27.68	110	70	2	1	2	1	1	1	1	5	2
25	1	52	1	25	23.88	140	97	2	2	2	2	2	1	2	7	1
26	1	49	1	25	28.72	114	70	1	1	2	1	1	1	1	5	1
27	1	54	1	22	22.58	100	60	2	2	1	1	1	1	1	4	1
28	1	48	1	16	23.44	100	79	2	2	1	1	1	1	1	17	2
29	1	32	1	18	29.28	110	80	1	1	1	2	1	1	1	6	1
30	1	35	2	18	31.25	100	70	1	1	2	1	2	2	1	6	1
31	1	78	2	24	miss	95	55	1	2	1	2	1	1	2	8	1
32	1	68	2	22	27.34	131	62	1	2	1	2	1	1	1	3	1
33	1	59	2	20	29.38	140	80	1	1	1	1	1	1	1	9	1
34	1	40	1	18	28.08	85	60	1	2	2	2	1	1	1	5	1
35	1	43	1	25	29.75	110	70	1	2	2	2	1	1	2	4	1
36	1	60	2	21	29.69	100	60	2	2	1	2	1	1	2	5	1
37	1	45	2	18	29.30	150	90	2	2	1	2	1	1	1	5	1
38	1	31	1	22	31.24	100	80	2	1	2	2	2	1	1	4	1
39	1	18	2	20	25.95	120	80	2	1	2	1	2	2	1	6	1
40	1	55	1	26	28.73	156	85	2	2	2	1	1	2	1	6	1
41	1	48	1	20	29.41	120	80	1	1	2	2	2	1	1	5	1
42	1	47	2	20	23.44	140	80	2	2	1	2	1	1	2	6	1
43	1	39	1	18	28.34	130	80	2	1	2	2	2	1	1	5	1
44	1	62	2	18	44.82	140	80	1	1	1	2	1	1	1	1	2
45	1	49	1	18	32.99	120	80	2	2	2	2	1	1	1	3	1
46	1	34	1	24	29.32	140	80	1	1	1	1	1	1	2	4	1
47	1	35	2	20	22.84	110	60	1	1	2	1	1	1	1	4	1

EXCLI Journal 2021;20:444-453 – ISSN 1611-2156
Received: January 16, 2021, accepted: February 16, 2021, published: February 22, 2021

48	1	45	2	18	33.06	150	90	1	2	1	2	1	1	1	5	1
49	1	54	1	miss	27.43	miss	miss	1	2	2	1	2	1	1	4	1
50	1	35	1	26	23.88	129	90	2	2	1	2	2	1	1	6	1
51	1	34	2	17	28.34	110	70	2	1	2	2	2	1	1	5	1
52	1	45	2	18	29.04	100	60	2	2	2	2	2	2	1	12	1
53	1	51	1	19	29.01	140	90	1	1	2	1	1	1	1	7	1
54	1	53	2	20	28.93	140	90	2	1	1	1	1	2	1	5	1
55	1	25	2	20	27.34	130	80	2	1	2	2	2	2	1	5	1
56	1	44	1	22	27.55	128	77	2	1	2	2	1	1	1	4	1
57	1	33	1	26	30.42	127	80	2	2	2	1	1	1	2	4	1
58	1	62	1	25	24.22	125	80	2	1	1	1	1	1	1	8	1
59	1	39	1	22	24.98	125	80	2	1	1	2	1	1	2	8	2
60	1	45	2	27	30.48	131	100	1	2	1	1	1	1	2	5	1
61	1	48	2	22	35.16	130	70	1	2	2	2	1	1	2	18	1
62	1	47	1	27	27.55	128	75	2	2	2	1	1	1	1	10	1
63	1	35	1	26	28.23	135	88	1	2	2	2	1	1	2	6	1
64	1	43	1	20	21.08	100	60	1	1	2	2	1	2	2	5	1
65	1	41	1	23	26.30	110	70	1	2	2	2	1	1	1	8	1
66	1	27	1	23	25.47	120	80	2	1	2	2	1	2	1	7	1
67	1	30	2	20	28.04	110	70	1	2	2	1	2	2	1	4	1
68	1	72	1	22	35.16	140	80	2	2	1	2	1	1	1	10	1
69	1	81	2	22	30.49	120	80	2	1	1	2	1	1	2	6	1
70	1	62	1	22	27.43	120	70	2	2	1	1	1	1	2	6	1
71	1	72	1	23	25.31	160	90	1	2	2	1	2	1	1	miss	1
72	1	70	2	25	24.91	118	80	2	1	1	2	1	1	1	10	1
73	1	57	2	22	26.22	127	86	2	2	1	2	1	1	2	11	1
74	1	33	2	20	25.08	125	80	1	2	2	1	1	2	1	4	1
75	1	37	2	22	27.55	141	85	2	1	1	1	1	1	1	3	1
76	1	46	2	20	24.22	120	80	2	1	2	1	2	1	2	4	1
77	1	45	1	18	29.07	110	70	1	2	2	2	1	1	1	6	1
78	1	34	2	23	26.51	115	80	2	2	2	1	2	1	1	8	1
79	1	73	2	23	29.01	140	95	1	2	2	2	1	1	1	5	1
80	1	43	1	20	18.83	100	60	1	2	2	1	1	1	2	6	1
81	1	56	2	22	23.01	150	90	1	2	1	1	1	1	2	7	1
82	1	63	2	24	35.56	130	90	2	1	2	2	1	1	1	6	1
83	1	65	2	22	24.22	120	80	2	1	1	1	1	1	1	9	1
84	1	74	1	20	23.60	170	100	2	2	2	2	1	1	2	5	1
85	1	29	1	22	29.41	120	70	1	2	2	1	1	1	1	5	1
86	1	58	2	18	28.03	130	100	2	1	1	2	1	1	2	7	1
87	1	60	1	19	23.38	131	100	1	2	2	1	1	1	1	4	1
88	1	53	2	19	21.31	155	100	1	1	1	1	1	2	1	4	1
89	1	45	2	18	31.22	120	70	1	1	1	2	1	2	1	7	1
90	1	56	2	17	34.67	100	60	2	1	1	2	1	1	1	6	1
91	1	69	1	18	28.39	110	70	2	2	2	1	1	1	1	1	1
92	1	66	1	25	30.12	120	80	2	2	1	2	1	1	1	4	1
93	1	35	2	21	24.68	125	80	1	2	1	1	1	1	1	5	1
94	1	72	1	18	29.05	130	90	1	1	1	2	1	1	1	2	1
95	1	79	2	20	29.00	130	80	1	1	2	2	2	1	1	3	2
96	1	40	2	25	33.87	110	80	1	1	2	1	1	1	1	5	1
97	1	51	2	22	25.95	130	80	1	2	2	2	1	1	2	6	1
98	1	28	2	25	27.55	143	85	1	1	2	1	1	1	1	5	1
99	1	32	1	20	28.96	110	80	1	1	1	1	1	1	1	1	1
100	1	53	2	19	29.69	180	100	1	1	2	1	1	1	1	5	1
101	1	61	2	21	28.84	180	100	1	2	2	2	2	1	1	5	1
102	1	60	1	20	24.28	130	80	1	2	2	2	1	1	1	9	1
103	1	51	1	20	26.83	110	70	2	2	1	2	1	1	1	4	1

EXCLI Journal 2021;20:444-453 – ISSN 1611-2156
Received: January 16, 2021, accepted: February 16, 2021, published: February 22, 2021

104	1	34	1	22	22.72	110	70	1	2	2	2	1	2	1	9	1
105	1	65	2	18	35.16	130	80	1	2	2	2	1	1	1	7	1
106	1	71	2	20	27.43	150	90	1	1	1	2	1	1	1	6	1
107	1	44	1	20	23.78	110	80	1	1	2	1	2	1	1	5	1
108	1	30	2	22	20.55	120	80	2	2	2	2	2	2	1	5	1
109	1	33	2	22	24.03	100	60	2	2	2	2	2	2	1	4	1
110	1	64	2	23	24.77	135	85	2	2	2	2	2	1	2	6	1
111	1	41	2	25	23.38	120	75	2	2	2	2	2	1	1	4	1
112	1	69	2	20	31.25	130	80	1	2	1	1	1	1	1	4	2
113	1	47	1	26	24.69	120	73	2	2	1	2	1	1	2	2	2
114	1	59	2	25	37.58	120	75	1	2	2	2	2	1	1	5	2
115	1	75	1	26	28.58	130	80	2	2	2	2	2	1	1	12	1
116	1	74	1	23	24.62	95	65	1	1	1	1	1	1	1	10	2
117	1	74	1	26	22.94	130	80	2	2	1	1	2	1	1	6	1
118	1	43	1	18	24.54	110	70	1	2	2	1	2	1	1	10	1
119	1	40	2	20	25.10	120	80	1	2	1	1	2	1	1	4	1
120	1	66	1	20	23.12	130	80	1	2	2	1	2	1	1	7	1
121	1	64	1	22	23.08	130	80	2	2	2	1	2	1	2	6	1
122	1	56	1	19	22.86	130	80	2	1	1	1	1	2	1	7	1
123	1	35	1	25	22.53	113	70	1	2	2	1	2	1	1	6	1
124	1	41	1	22	26.89	110	70	1	1	2	1	1	2	1	5	1
125	1	35	1	22	23.77	125	80	2	2	1	1	1	1	1	3	1
126	1	59	1	23	28.34	111	59	1	2	1	1	1	1	1	7	1
127	1	43	1	20	25.76	140	90	1	1	2	1	2	1	2	5	1
128	1	58	2	22	26.84	130	80	2	2	2	2	2	1	1	9	1
129	1	49	1	26	23.08	150	97	2	1	2	2	2	1	1	6	1
130	1	58	1	22	21.46	140	90	2	2	2	1	2	1	1	8	1
131	1	31	2	20	28.04	110	70	1	1	2	1	2	1	1	6	1
132	1	57	2	22	29.64	110	70	1	1	2	1	2	2	1	5	1
133	1	68	2	25	25.99	129.	80	1	2	2	1	2	1	1	5	1
134	1	50	1	20	26.12	120	80	2	1	2	2	2	1	1	5	1
135	1	40	2	26	27.78	110	60	1	2	2	1	2	1	1	4	1
136	1	67	2	18	27.55	130	80	1	2	1	2	1	1	2	5	1
137	1	57	2	22	25.83	135	91	2	1	2	2	2	1	1	6	1
138	1	43	2	24	27.25	145	80	2	2	2	1	2	1	1	4	1
139	1	61	1	18	25.28	140	80	2	2	1	2	2	1	1	9	1
140	1	44	1	22	26.12	129	75	1	2	2	1	2	1	1	7	1
141	1	31	2	24	21.08	120	70	2	2	2	1	2	1	1	6	1
142	2	60	2	20	24.91	130	80	2	2	1	2	1	1	1	4	1
143	2	60	2	20	35.94	139	80	1	2	1	2	2	2	1	15	2
144	2	34	1	25	33.83	130	90	2	2	2	2	1	1	1	7	1
145	2	67	1	26	27.55	150	110	1	2	1	2	2	1	1	1	2
146	2	52	2	23	32.42	120	80	1	2	2	1	1	1	1	6	1
147	2	59	1	25	31.59	142	83	1	2	1	2	1	1	1	8	2
148	2	73	1	25	26.12	138	91	1	2	2	1	1	1	2	12	2
149	2	55	2	24	33.06	130	85	2	2	2	1	1	1	1	7	2
150	2	72	2	25	24.84	111	64	2	2	2	2	1	1	1	6	1
151	2	60	2	28	29.30	160	90	1	2	2	2	1	1	2	15	1
152	2	53	2	28	32.03	175	9	2	2	1	1	1	2	1	8	2
153	2	48	1	24	31.83	120	80	2	1	2	2	1	1	1	4	1
154	2	72	2	23	29.41	139	83	1	2	2	1	1	1	1	7	1
155	2	67	2	20	25.39	110	70	2	2	1	2	1	1	2	6	1
156	2	66	2	22	28.20	170	100	1	1	2	2	2	1	2	4	1
157	2	60	2	22	30.47	120	70	1	2	2	2	1	1	2	4	2
158	2	57	1	18	30.08	165	95	1	1	2	1	1	1	1	6	1
159	2	39	2	miss	27.85	miss	miss	1	2	2	1	1	1	2	7	2

160	2	76	2	18	31.89	110	80	2	1	2	1	1	1	2	8	1
161	2	56	1	26	26.37	125	75	2	2	1	2	1	1	1	2	2
162	2	44	2	20	28.30	120	70	2	1	2	1	2	1	1	6	1
163	2	77	1	27	27.34	178	90	1	2	1	2	1	1	1	3	2
164	2	79	2	24	31.22	130	80	2	2	2	1	1	1	1	4	2
165	2	63	1	20	27.64	110	70	2	1	1	1	1	1	1	5	2
166	2	31	1	19	32.27	100	60	2	1	2	1	1	2	1	7	1
167	2	60	1	18	29.39	110	70	1	2	2	1	1	1	1	3	2
168	2	46	2	20	23.44	140	80	2	2	2	2	2	1	1	5	1
169	2	43	2	24	44.08	145	80	2	1	2	1	2	1	1	6	1
170	2	48	2	28	30.11	110	70	1	1	2	1	1	1	1	12	1
171	2	62	1	18	28.58	110	70	1	2	1	2	2	1	1	16	1
172	2	61	1	26	26.64	120	75	2	2	1	1	1	1	1	2	1
173	2	55	1	26	29.76	140	82	1	2	2	2	1	1	1	11	1
174	2	60	2	24	31.99	179	100	1	2	2	2	1	1	1	20	2
175	2	47	1	25	30.12	110	75	1	2	1	2	1	1	2	12	2
176	2	69	1	20	29.07	110	70	1	2	1	1	1	1	1	5	1
177	2	66	2	miss	miss	miss	miss	1	2	2	1	1	1	2	3	1
178	2	75	2	22	miss	110	70	1	2	1	1	1	1	1	1	2
179	2	51	1	26	28.73	159	86	1	2	2	1	1	1	1	5	1
180	2	43	2	18	32.87	100	70	2	2	1	2	1	1	2	6	1
181	2	55	2	20	28.01	110	70	1	1	1	1	1	1	1	2	2
182	2	73	2	26	28.54	102	63	1	2	2	2	1	1	1	2	2
183	2	70	2	29	23.11	104	68	2	2	1	1	1	1	1	4	1
184	2	71	1	26	25.88	140	80	1	2	1	2	1	1	1	4	2
185	2	51	1	20	29.36	163	90	1	1	2	1	1	1	1	4	1
186	2	78	1	26	20.99	116	70	2	2	1	1	1	1	1	6	1
187	2	76	1	22	24.97	110	70	2	1	2	1	2	2	1	8	1
188	2	55	1	24	27.76	120	78	2	1	2	2	1	1	1	4	1
189	2	58	1	20	23.57	125	80	2	1	2	2	1	1	1	4	2
190	2	48	1	23	36.85	135	80	2	1	2	2	1	1	2	5	1
191	2	80	1	24	35.16	190	110	1	1	1	1	1	1	2	1	1
192	2	63	1	24	26.53	127	77	1	2	2	2	2	1	1	7	1
193	2	74	1	24	34.60	160	80	1	2	1	2	1	1	2	6	1
194	2	51	2	19	26.08	120	80	1	1	1	1	2	1	1	6	1
195	2	74	2	35	29.38	126	86	2	2	2	1	1	1	1	8	2
196	2	72	1	26	25.95	130	90	2	1	1	2	1	1	1	13	2
197	2	64	1	18	28.07	135	68	1	2	2	2	1	1	2	6	1
198	2	66	1	23	31.24	145	90	2	1	1	2	1	1	2	5	1
199	2	74	1	26	32.87	160	95	2	2	2	2	2	1	2	6	1
200	2	58	2	30	32.39	130	85	2	2	2	2	1	1	2	8	2
201	2	68	2	miss	31.25	miss	miss	1	1	2	2	2	2	1	13	2
202	2	48	2	miss	35.16	miss	miss	2	2	2	2	2	1	1	10	1
203	2	68	2	miss	33.30	miss	miss	1	1	2	2	2	1	1	7	1
204	2	68	2	miss	34.13	miss	miss	2	1	2	2	2	1	1	7	1
205	2	61	1	miss	33.20	miss	miss	2	2	2	2	2	1	2	8	1
206	2	76	1	miss	27.99	miss	miss	2	2	2	1	1	1	1	3	2
207	2	79	1	miss	32.32	miss	miss	1	2	2	1	1	1	1	3	2
208	2	68	2	miss	31.24	miss	miss	2	2	2	2	2	1	1	8	2
209	2	62	1	miss	27.55	miss	miss	1	2	1	2	1	1	1	12	1
210	2	69	1	miss	26.23	miss	miss	2	2	2	2	1	1	2	9	1
211	2	73	1	miss	24.22	miss	miss	2	2	2	1	2	1	2	9	2
212	2	60	2	miss	34.60	miss	miss	2	2	1	2	2	1	2	20	2
213	2	74	2	miss	30.04	miss	miss	2	2	2	2	2	1	1	10	2
214	2	68	1	miss	42.45	miss	miss	1	2	2	2	2	1	1	8	2
215	2	42	1	miss	28.06	miss	miss	1	2	2	2	1	2	1	8	1

EXCLI Journal 2021;20:444-453 – ISSN 1611-2156
Received: January 16, 2021, accepted: February 16, 2021, published: February 22, 2021

216	2	60	2	miss	25.39	miss	miss	1	2	2	2	1	1	2	14	2
217	2	65	1	miss	26.83	miss	miss	1	1	2	2	1	1	2	21	2
218	2	66	2	miss	24.22	miss	miss	1	1	1	2	1	1	2	10	1
219	2	59	1	miss	33.30	miss	miss	1	1	2	2	1	1	1	9	2
220	2	55	1	miss	27.68	miss	miss	1	1	2	2	1	1	1	13	2
221	2	78	2	miss	32.37	miss	miss	2	1	2	2	1	1	2	13	2
222	2	79	2	miss	27.06	miss	miss	1	2	2	1	1	1	1	11	2
223	2	72	2	miss	34.60	miss	miss	2	1	2	1	2	2	2	7	2
224	2	66	1	miss	31.22	miss	miss	2	1	2	1	1	1	1	7	1
225	2	71	1	miss	26.78	miss	miss	1	2	1	2	1	1	1	3	1
226	2	62	1	miss	33.66	miss	miss	1	2	2	2	1	1	2	12	1
227	2	61	1	miss	25.00	miss	miss	1	2	2	1	1	1	1	19	1
228	2	78	2	miss	35.25	miss	miss	2	1	2	1	2	1	2	4	2
229	2	52	1	miss	33.79	miss	miss	2	1	2	1	1	1	1	6	2
230	2	72	1	miss	30.74	miss	miss	2	1	2	2	1	1	1	5	1
231	2	46	2	miss	32.03	miss	miss	1	1	1	1	1	1	1	2	2
232	2	64	1	miss	36.20	miss	miss	2	1	2	2	2	1	1	1	2
233	2	60	2	miss	32.03	miss	miss	2	1	2	2	1	1	1	5	2
234	2	71	1	miss	30.45	miss	miss	2	1	2	2	1	1	2	5	2
235	2	60	1	miss	25.42	miss	miss	2	1	1	1	1	1	1	2	1
236	2	73	2	miss	32.81	miss	miss	2	1	2	2	1	1	1	9	1
237	2	70	1	miss	32.32	miss	miss	2	1	2	2	1	1	1	8	2
238	2	miss	2	miss	44.01	miss	miss	2	2	1	1	1	1	1	5	1
239	2	65	1	miss	34.53	miss	miss	2	1	2	1	2	1	1	5	1
240	2	70	2	miss	33.33	miss	miss	1	1	1	2	1	1	2	7	1
241	2	65	2	miss	33.59	miss	miss	1	1	2	2	1	1	1	4	1
242	2	44	1	miss	40.12	miss	miss	1	1	2	1	2	1	2	4	1
243	2	67	1	miss	29.04	miss	miss	2	1	1	2	1	1	1	4	1
244	2	53	1	miss	31.35	miss	miss	2	1	2	2	1	1	1	14	1
245	2	53	2	miss	31.89	miss	miss	1	1	2	1	1	1	2	11	2
246	2	45	1	miss	29.41	miss	miss	2	1	2	1	1	1	2	5	1
247	2	49	1	miss	35.16	miss	miss	2	1	2	1	1	1	1	18	2
248	2	76	1	miss	28.34	miss	miss	2	1	2	2	1	1	1	3	2
249	2	34	1	miss	34.60	miss	miss	2	1	2	2	1	1	2	1	2
250	2	64	1	miss	29.67	miss	miss	2	1	2	2	1	1	1	5	2
251	2	77	2	miss	29.38	miss	miss	1	1	2	1	1	1	2	5	2
252	2	54	2	miss	35.16	miss	miss	1	1	2	1	1	1	2	2	2
253	2	65	1	miss	26.12	miss	miss	2	1	2	1	1	1	1	1	2
254	2	65	2	miss	27.06	miss	miss	2	2	2	2	2	1	2	18	2
255	2	69	2	miss	31.25	miss	miss	1	1	2	2	2	1	1	2	2
256	2	63	1	miss	27.76	miss	miss	2	1	2	1	2	1	1	6	2
257	2	73	1	miss	31.59	miss	miss	1	2	2	2	2	1	1	2	2
258	2	60	2	miss	31.22	miss	miss	1	1	2	2	1	1	2	6	1
259	2	57	2	miss	29.76	miss	miss	1	1	2	2	1	1	1	3	1
260	2	75	2	miss	35.16	miss	miss	1	1	2	2	2	1	1	13	2
261	2	64	1	miss	25.71	miss	miss	1	1	2	2	1	1	1	14	1
262	2	66	2	miss	29.30	miss	miss	1	1	2	2	1	1	1	14	1
263	2	61	2	miss	28.12	miss	miss	2	1	2	2	1	2	2	14	1
264	2	78	1	miss	24.62	miss	miss	2	1	2	2	2	1	1	14	1
265	2	54	1	miss	27.77	miss	miss	1	2	2	1	1	1	1	14	1
266	2	79	2	miss	46.87	miss	miss	2	1	2	1	1	1	1	12	1
267	2	80	1	miss	miss	miss	miss	1	1	1	1	2	1	1	14	2
268	2	69	2	miss	31.22	miss	miss	2	1	2	2	2	1	2	15	2

ID: Identity number, M: Male, F: Female, BMI: Body mass index, SBP: Systolic blood pressure, DBP: Diastolic blood pressure, GI: Gastrointestinal, LOS: Length of hospital stay, ICU: Intensive care unit

EXCLI Journal 2021;20:444-453 – ISSN 1611-2156
Received: January 16, 2021, accepted: February 16, 2021, published: February 22, 2021

Supplementary Table 2: Raw data of comorbidities of diabetic and non-diabetic patients with COVID-19 which is shown in Table 1 of the manuscript.

ID	Chronic pulmonary disease 1: No 2: Yes	Coronary heart disease 1: No 2: Yes	Hypertension 1: No 2: Yes	Chronic renal diseases 1: No 2: Yes	Chronic liver disease 1: No 2: Yes	Obesity 1: No 2: Yes	Brain disease 1: No 2: Yes
1	1	1	1	1	1	miss	1
2	1	2	1	1	1	miss	1
3	1	2	2	1	1	1	1
4	2	2	2	1	1	1	1
5	1	1	1	1	1	1	1
6	1	1	1	1	1	miss	1
7	1	1	1	1	1	1	1
8	1	1	1	1	1	1	1
9	1	1	2	1	1	miss	1
10	1	2	2	1	1	2	1
11	1	1	1	1	1	1	2
12	1	1	1	1	1	1	1
13	1	2	1	1	1	1	1
14	2	1	1	1	1	1	1
15	1	2	2	1	1	1	1
16	1	1	1	1	1	2	1
17	1	1	1	1	1	1	1
18	1	1	1	1	1	1	1
19	1	2	2	1	1	1	1
20	1	1	1	1	1	1	1
21	1	2	1	1	1	2	1
22	2	1	2	1	1	1	1
23	2	2	2	1	1	2	1
24	2	1	1	1	1	1	1
25	1	1	2	1	1	1	1
26	1	2	1	1	1	1	1
27	1	1	1	1	1	1	1
28	1	1	1	1	1	1	2
29	1	1	1	1	1	1	1
30	1	1	1	1	1	2	1
31	1	2	2	1	1	miss	1
32	2	2	1	1	1	1	1
33	1	1	2	1	1	1	1
34	1	1	1	1	1	1	1
35	1	1	1	1	1	1	1
36	1	2	1	1	1	1	1
37	1	1	1	1	1	1	1
38	1	1	1	1	1	2	1
39	1	1	1	1	1	1	1
40	1	1	1	1	1	1	1
41	1	1	1	1	1	1	1
42	1	1	1	1	1	1	1
43	1	1	1	1	1	1	1
44	1	2	2	1	1	2	1
45	1	1	1	1	1	2	1
46	1	1	1	1	1	1	1
47	1	1	1	1	1	1	1
48	1	1	2	2	1	2	1
49	1	2	1	1	1	1	1
50	2	1	1	1	1	1	1
51	1	1	1	1	1	1	1
52	1	1	1	1	1	1	1
53	1	1	1	1	1	1	1
54	1	1	2	1	1	1	1
55	1	1	1	1	1	1	1
56	1	1	1	1	1	1	1
57	1	1	1	1	1	2	1
58	1	1	1	1	1	1	2
59	1	1	1	1	1	1	1
60	2	2	1	1	1	2	1
61	1	1	1	1	1	2	1
62	1	1	1	1	1	1	1
63	1	1	1	1	1	1	1
64	1	1	1	1	1	1	1
65	1	1	1	1	1	1	1
66	1	1	1	1	1	1	1
67	1	1	1	1	1	1	1
68	1	1	2	1	1	2	1
69	1	1	2	1	1	2	1
70	1	1	1	1	1	1	1
71	1	1	1	1	1	1	1
72	1	1	1	1	1	1	1
73	1	2	2	1	1	1	1
74	1	1	1	1	1	1	1
75	1	1	1	1	1	1	1
76	1	1	1	1	1	1	1

77	1	1	1	1	1	1	1
78	1	1	1	1	1	1	1
79	1	1	1	1	1	1	1
80	1	1	1	1	1	1	1
81	1	1	2	1	1	1	1
82	1	1	2	1	1	2	1
83	1	2	2	1	1	1	1
84	2	2	2	1	1	1	1
85	1	1	1	1	1	1	1
86	1	2	2	1	1	1	1
87	1	1	1	1	1	1	1
88	1	1	1	1	1	1	1
89	1	1	1	1	1	2	1
90	1	1	1	1	1	2	1
91	1	2	1	1	1	1	1
92	1	1	1	1	1	2	1
93	2	1	1	1	1	1	1
94	1	2	2	1	1	1	1
95	1	2	2	2	1	1	1
96	1	1	1	1	1	2	1
97	miss	miss	miss	miss	miss	1	miss
98	2	1	1	1	1	1	1
99	1	1	1	1	1	1	1
100	1	1	2	1	1	1	1
101	1	1	1	1	1	1	1
102	1	2	2	1	1	1	1
103	1	1	1	1	1	1	1
104	1	1	1	1	1	1	1
105	1	1	2	1	1	2	1
106	1	1	2	1	1	1	1
107	1	1	1	1	1	1	1
108	1	1	1	1	1	1	1
109	1	1	1	1	1	1	1
110	1	1	1	1	1	1	1
111	1	1	1	1	1	1	1
112	1	1	2	1	1	2	1
113	1	1	1	1	1	1	1
114	1	1	1	1	1	2	1
115	1	1	1	1	1	1	1
116	1	2	1	1	1	1	2
117	1	2	2	1	1	1	2
118	1	1	1	1	1	1	1
119	1	1	1	1	1	1	1
120	1	1	1	1	1	1	1
121	1	1	1	1	1	1	1
122	1	1	1	1	1	1	1
123	1	1	1	1	1	1	2
124	1	1	1	1	1	1	1
125	1	1	1	1	1	1	1
126	2	2	1	1	1	1	2
127	1	1	1	1	1	1	1
128	1	1	2	1	1	1	1
129	1	1	1	1	1	1	1
130	1	1	2	1	1	1	1
131	1	1	1	1	1	1	1
132	1	1	1	1	1	1	1
133	1	2	2	1	1	1	1
134	1	1	1	1	1	1	1
135	1	1	1	1	1	1	1
136	1	1	2	1	1	1	1
137	1	1	1	1	1	1	1
138	1	1	1	1	1	1	1
139	2	1	2	1	1	1	1
140	1	1	1	1	1	1	1
141	1	1	1	1	1	1	1
142	2	1	2	1	1	1	1
143	1	1	1	1	1	2	1
144	1	1	2	1	1	2	1
145	2	1	2	1	1	1	1
146	1	1	1	1	1	2	1
147	1	2	2	1	1	2	1
148	1	2	2	1	1	1	1
149	1	1	1	1	1	2	1
150	1	2	2	2	1	1	1
151	1	1	2	1	1	1	1
152	1	2	1	1	1	2	1
153	1	1	1	1	1	2	1
154	1	1	2	1	1	1	1
155	1	1	2	1	1	1	1
156	1	1	2	1	1	1	1
157	2	2	1	1	1	2	1
158	1	1	1	1	1	2	1
159	1	1	1	1	1	1	1
160	1	2	2	1	1	2	1
161	1	2	2	1	1	1	1

EXCLI Journal 2021;20:444-453 – ISSN 1611-2156
Received: January 16, 2021, accepted: February 16, 2021, published: February 22, 2021

162	1	1	1	1	1	1	1
163	2	2	2	1	1	1	1
164	2	1	2	1	1	2	1
165	1	1	1	1	1	1	1
166	1	1	1	1	1	2	1
167	1	2	1	1	1	1	1
168	1	1	1	1	1	1	2
169	1	1	1	1	1	2	1
170	1	1	2	1	1	2	1
171	1	1	2	1	1	1	1
172	1	1	2	2	1	1	1
173	1	1	2	1	1	1	1
174	1	1	2	2	1	2	1
175	1	1	2	2	1	2	1
176	1	1	2	1	1	1	1
177	1	1	2	2	1	miss	1
178	1	2	2	1	1	miss	1
179	1	2	1	1	1	1	1
180	1	1	1	1	1	2	1
181	2	1	1	2	1	1	1
182	1	2	1	1	1	1	1
183	1	2	2	1	1	1	1
184	2	2	2	1	1	1	1
185	1	1	1	1	1	1	1
186	1	2	1	1	1	1	1
187	1	1	1	1	1	1	1
188	1	1	1	1	1	1	1
189	1	2	2	1	1	1	1
190	1	1	2	1	1	2	1
191	1	1	2	1	2	2	1
192	1	2	2	1	1	1	1
193	1	2	2	1	1	2	1
194	1	miss	miss	miss	miss	1	miss
195	1	1	2	1	1	1	1
196	1	2	1	1	1	1	1
197	1	2	2	2	1	1	1
198	1	1	2	1	1	2	1
199	1	2	2	1	1	2	1
200	1	1	1	1	1	2	1
201	1	1	1	1	1	2	1
202	1	1	1	1	1	2	1
203	2	2	2	1	1	2	1
204	1	2	2	1	1	2	1
205	2	1	2	1	1	2	1
206	1	1	1	1	1	1	1
207	1	2	1	1	1	2	1
208	1	2	2	1	1	2	1
209	1	1	1	1	1	1	1
210	1	1	1	1	1	1	1
211	1	2	2	1	1	1	1
212	1	1	2	1	1	2	1
213	1	2	2	1	1	2	1
214	1	2	2	1	1	2	1
215	1	1	1	1	1	1	1
216	1	1	2	1	1	1	1
217	2	1	2	1	1	1	1
218	1	1	2	1	1	1	1
219	2	2	1	1	1	2	1
220	1	2	1	1	1	1	1
221	2	2	2	1	1	2	1
222	1	2	2	1	1	1	1
223	1	2	2	1	1	2	1
224	1	1	2	1	1	2	1
225	1	1	2	1	1	1	1
226	1	2	1	1	1	2	1
227	1	1	2	1	1	1	1
228	1	2	2	1	1	2	1
229	1	1	2	2	1	2	1
230	1	1	1	1	1	2	1
231	1	2	2	1	1	2	1
232	1	2	2	1	1	2	1
233	2	2	2	1	1	2	1
234	1	1	2	1	1	2	1
235	1	2	1	1	1	1	1
236	2	1	2	1	1	2	1
237	1	2	2	1	1	2	1
238	1	1	1	1	1	2	1
239	1	1	2	1	1	2	1
240	1	2	2	1	1	2	1
241	1	1	1	1	1	2	1
242	1	1	1	1	1	2	1
243	1	1	1	1	1	1	1
244	1	1	1	2	1	2	1
245	1	1	2	1	1	2	1
246	1	1	1	1	1	1	1

247	1	1	1	1	1	2	1
248	1	2	2	1	1	1	1
249	1	1	1	1	1	2	1
250	1	2	2	1	1	1	1
251	1	1	1	1	1	1	1
252	1	1	1	1	1	2	1
253	1	2	2	1	1	1	1
254	1	2	2	1	1	1	1
255	1	1	1	1	1	2	1
256	1	2	1	1	1	1	1
257	1	1	1	1	1	2	1
258	1	2	2	1	1	2	1
259	1	1	2	1	1	1	1
260	1	1	2	1	1	2	1
261	1	1	2	1	1	1	1
262	1	2	2	1	1	1	1
263	1	1	2	1	1	1	1
264	1	1	2	1	1	1	1
265	1	1	1	1	1	1	1
266	1	1	2	1	1	2	1
267	1	1	1	1	1	miss	1
268	1	2	2	1	1	2	1

EXCLI Journal 2021;20:444-453 – ISSN 1611-2156
Received: January 16, 2021, accepted: February 16, 2021, published: February 22, 2021

Supplementary Table 3: Raw data of laboratory examination of diabetic and non-diabetic patients with COVID-19 which is shown in Table 1 of the manuscript.

ID	Chronic pulmonary disease 1: No 2: Yes	Coronary heart disease 1: No 2: Yes	Hypertension 1: No 2: Yes	Chronic renal diseases 1: No 2: Yes	Chronic liver disease 1: No 2: Yes	Obesity 1: No 2: Yes	Brain disease 1: No 2: Yes
1	1	1	1	1	1	miss	1
2	1	2	1	1	1	miss	1
3	1	2	2	1	1	1	1
4	2	2	2	1	1	1	1
5	1	1	1	1	1	1	1
6	1	1	1	1	1	miss	1
7	1	1	1	1	1	1	1
8	1	1	1	1	1	1	1
9	1	1	2	1	1	miss	1
10	1	2	2	1	1	2	1
11	1	1	1	1	1	1	2
12	1	1	1	1	1	1	1
13	1	2	1	1	1	1	1
14	2	1	1	1	1	1	1
15	1	2	2	1	1	1	1
16	1	1	1	1	1	2	1
17	1	1	1	1	1	1	1
18	1	1	1	1	1	1	1
19	1	2	2	1	1	1	1
20	1	1	1	1	1	1	1
21	1	2	1	1	1	2	1
22	2	1	2	1	1	1	1
23	2	2	2	1	1	2	1
24	2	1	1	1	1	1	1
25	1	1	2	1	1	1	1
26	1	2	1	1	1	1	1
27	1	1	1	1	1	1	1
28	1	1	1	1	1	1	2
29	1	1	1	1	1	1	1
30	1	1	1	1	1	2	1
31	1	2	2	1	1	miss	1
32	2	2	1	1	1	1	1
33	1	1	2	1	1	1	1
34	1	1	1	1	1	1	1
35	1	1	1	1	1	1	1
36	1	2	1	1	1	1	1
37	1	1	1	1	1	1	1
38	1	1	1	1	1	2	1
39	1	1	1	1	1	1	1
40	1	1	1	1	1	1	1
41	1	1	1	1	1	1	1
42	1	1	1	1	1	1	1
43	1	1	1	1	1	1	1
44	1	2	2	1	1	2	1
45	1	1	1	1	1	2	1
46	1	1	1	1	1	1	1
47	1	1	1	1	1	1	1
48	1	1	2	2	1	2	1
49	1	2	1	1	1	1	1
50	2	1	1	1	1	1	1
51	1	1	1	1	1	1	1
52	1	1	1	1	1	1	1
53	1	1	1	1	1	1	1
54	1	1	2	1	1	1	1
55	1	1	1	1	1	1	1
56	1	1	1	1	1	1	1
57	1	1	1	1	1	2	1
58	1	1	1	1	1	1	2
59	1	1	1	1	1	1	1
60	2	2	1	1	1	2	1
61	1	1	1	1	1	2	1
62	1	1	1	1	1	1	1
63	1	1	1	1	1	1	1
64	1	1	1	1	1	1	1
65	1	1	1	1	1	1	1
66	1	1	1	1	1	1	1
67	1	1	1	1	1	1	1
68	1	1	2	1	1	2	1
69	1	1	2	1	1	2	1
70	1	1	1	1	1	1	1
71	1	1	1	1	1	1	1
72	1	1	1	1	1	1	1
73	1	2	2	1	1	1	1
74	1	1	1	1	1	1	1
75	1	1	1	1	1	1	1
76	1	1	1	1	1	1	1

EXCLI Journal 2021;20:444-453 – ISSN 1611-2156
Received: January 16, 2021, accepted: February 16, 2021, published: February 22, 2021

77	1	1	1	1	1	1	1
78	1	1	1	1	1	1	1
79	1	1	1	1	1	1	1
80	1	1	1	1	1	1	1
81	1	1	2	1	1	1	1
82	1	1	2	1	1	2	1
83	1	2	2	1	1	1	1
84	2	2	2	1	1	1	1
85	1	1	1	1	1	1	1
86	1	2	2	1	1	1	1
87	1	1	1	1	1	1	1
88	1	1	1	1	1	1	1
89	1	1	1	1	1	2	1
90	1	1	1	1	1	2	1
91	1	2	1	1	1	1	1
92	1	1	1	1	1	2	1
93	2	1	1	1	1	1	1
94	1	2	2	1	1	1	1
95	1	2	2	2	1	1	1
96	1	1	1	1	1	2	1
97	miss	miss	miss	miss	miss	1	miss
98	2	1	1	1	1	1	1
99	1	1	1	1	1	1	1
100	1	1	2	1	1	1	1
101	1	1	1	1	1	1	1
102	1	2	2	1	1	1	1
103	1	1	1	1	1	1	1
104	1	1	1	1	1	1	1
105	1	1	2	1	1	2	1
106	1	1	2	1	1	1	1
107	1	1	1	1	1	1	1
108	1	1	1	1	1	1	1
109	1	1	1	1	1	1	1
110	1	1	1	1	1	1	1
111	1	1	1	1	1	1	1
112	1	1	2	1	1	2	1
113	1	1	1	1	1	1	1
114	1	1	1	1	1	2	1
115	1	1	1	1	1	1	1
116	1	2	1	1	1	1	2
117	1	2	2	1	1	1	2
118	1	1	1	1	1	1	1
119	1	1	1	1	1	1	1
120	1	1	1	1	1	1	1
121	1	1	1	1	1	1	1
122	1	1	1	1	1	1	1
123	1	1	1	1	1	1	2
124	1	1	1	1	1	1	1
125	1	1	1	1	1	1	1
126	2	2	1	1	1	1	2
127	1	1	1	1	1	1	1
128	1	1	2	1	1	1	1
129	1	1	1	1	1	1	1
130	1	1	2	1	1	1	1
131	1	1	1	1	1	1	1
132	1	1	1	1	1	1	1
133	1	2	2	1	1	1	1
134	1	1	1	1	1	1	1
135	1	1	1	1	1	1	1
136	1	1	2	1	1	1	1
137	1	1	1	1	1	1	1
138	1	1	1	1	1	1	1
139	2	1	2	1	1	1	1
140	1	1	1	1	1	1	1
141	1	1	1	1	1	1	1
142	2	1	2	1	1	1	1
143	1	1	1	1	1	2	1
144	1	1	2	1	1	2	1
145	2	1	2	1	1	1	1
146	1	1	1	1	1	2	1
147	1	2	2	1	1	2	1
148	1	2	2	1	1	1	1
149	1	1	1	1	1	2	1
150	1	2	2	2	1	1	1
151	1	1	2	1	1	1	1
152	1	2	1	1	1	2	1
153	1	1	1	1	1	2	1
154	1	1	2	1	1	1	1
155	1	1	2	1	1	1	1
156	1	1	2	1	1	1	1
157	2	2	1	1	1	2	1
158	1	1	1	1	1	2	1
159	1	1	1	1	1	1	1
160	1	2	2	1	1	2	1
161	1	2	2	1	1	1	1

EXCLI Journal 2021;20:444-453 – ISSN 1611-2156
Received: January 16, 2021, accepted: February 16, 2021, published: February 22, 2021

162	1	1	1	1	1	1	1
163	2	2	2	1	1	1	1
164	2	1	2	1	1	2	1
165	1	1	1	1	1	1	1
166	1	1	1	1	1	2	1
167	1	2	1	1	1	1	1
168	1	1	1	1	1	1	2
169	1	1	1	1	1	2	1
170	1	1	2	1	1	2	1
171	1	1	2	1	1	1	1
172	1	1	2	2	1	1	1
173	1	1	2	1	1	1	1
174	1	1	2	2	1	2	1
175	1	1	2	2	1	2	1
176	1	1	2	1	1	1	1
177	1	1	2	2	1	miss	1
178	1	2	2	1	1	miss	1
179	1	2	1	1	1	1	1
180	1	1	1	1	1	2	1
181	2	1	1	2	1	1	1
182	1	2	1	1	1	1	1
183	1	2	2	1	1	1	1
184	2	2	2	1	1	1	1
185	1	1	1	1	1	1	1
186	1	2	1	1	1	1	1
187	1	1	1	1	1	1	1
188	1	1	1	1	1	1	1
189	1	2	2	1	1	1	1
190	1	1	2	1	1	2	1
191	1	1	2	1	2	2	1
192	1	2	2	1	1	1	1
193	1	2	2	1	1	2	1
194	1	miss	miss	miss	miss	1	miss
195	1	1	2	1	1	1	1
196	1	2	1	1	1	1	1
197	1	2	2	2	1	1	1
198	1	1	2	1	1	2	1
199	1	2	2	1	1	2	1
200	1	1	1	1	1	2	1
201	1	1	1	1	1	2	1
202	1	1	1	1	1	2	1
203	2	2	2	1	1	2	1
204	1	2	2	1	1	2	1
205	2	1	2	1	1	2	1
206	1	1	1	1	1	1	1
207	1	2	1	1	1	2	1
208	1	2	2	1	1	2	1
209	1	1	1	1	1	1	1
210	1	1	1	1	1	1	1
211	1	2	2	1	1	1	1
212	1	1	2	1	1	2	1
213	1	2	2	1	1	2	1
214	1	2	2	1	1	2	1
215	1	1	1	1	1	1	1
216	1	1	2	1	1	1	1
217	2	1	2	1	1	1	1
218	1	1	2	1	1	1	1
219	2	2	1	1	1	2	1
220	1	2	1	1	1	1	1
221	2	2	2	1	1	2	1
222	1	2	2	1	1	1	1
223	1	2	2	1	1	2	1
224	1	1	2	1	1	2	1
225	1	1	2	1	1	1	1
226	1	2	1	1	1	2	1
227	1	1	2	1	1	1	1
228	1	2	2	1	1	2	1
229	1	1	2	2	1	2	1
230	1	1	1	1	1	2	1
231	1	2	2	1	1	2	1
232	1	2	2	1	1	2	1
233	2	2	2	1	1	2	1
234	1	1	2	1	1	2	1
235	1	2	1	1	1	1	1
236	2	1	2	1	1	2	1
237	1	2	2	1	1	2	1
238	1	1	1	1	1	2	1
239	1	1	2	1	1	2	1
240	1	2	2	1	1	2	1
241	1	1	1	1	1	2	1
242	1	1	1	1	1	2	1
243	1	1	1	1	1	1	1
244	1	1	1	2	1	2	1
245	1	1	2	1	1	2	1
246	1	1	1	1	1	1	1

EXCLI Journal 2021;20:444-453 – ISSN 1611-2156
Received: January 16, 2021, accepted: February 16, 2021, published: February 22, 2021

247	1	1	1	1	1	2	1
248	1	2	2	1	1	1	1
249	1	1	1	1	1	2	1
250	1	2	2	1	1	1	1
251	1	1	1	1	1	1	1
252	1	1	1	1	1	2	1
253	1	2	2	1	1	1	1
254	1	2	2	1	1	1	1
255	1	1	1	1	1	2	1
256	1	2	1	1	1	1	1
257	1	1	1	1	1	2	1
258	1	2	2	1	1	2	1
259	1	1	2	1	1	1	1
260	1	1	2	1	1	2	1
261	1	1	2	1	1	1	1
262	1	2	2	1	1	1	1
263	1	1	2	1	1	1	1
264	1	1	2	1	1	1	1
265	1	1	1	1	1	1	1
266	1	1	2	1	1	2	1
267	1	1	1	1	1	miss	1
268	1	2	2	1	1	2	1

EXCLI Journal 2021;20:444-453 – ISSN 1611-2156
Received: January 16, 2021, accepted: February 16, 2021, published: February 22, 2021

Supplementary Table 4: Raw data of Chest CT-scan of diabetic and non-diabetic patients with COVID-19 which is shown in Table 1 of the manuscript.

ID	Unilateral lesion<br>1: No<br>2: Yes	Bilateral lesions<br>1: No<br>2: Yes	Hospital mortality<br>1: Death<br>2: Discharged
1	2	1	2
2	1	2	2
3	1	2	2
4	1	2	2
5	1	2	2
6	1	2	2
7	1	2	1
8	2	1	2
9	1	2	2
10	1	2	2
11	2	1	2
12	1	1	2
13	1	2	2
14	2	1	1
15	1	2	2
16	1	2	2
17	1	2	2
18	1	2	2
19	1	2	2
20	1	2	2
21	1	2	2
22	1	2	2
23	1	2	2
24	2	1	2
25	2	1	2
26	1	2	2
27	2	1	2
28	1	2	1
29	1	2	2
30	1	2	2
31	1	2	2
32	1	2	2
33	1	2	2
34	1	2	2
35	2	1	2
36	1	2	2
37	1	2	2
38	1	1	2
39	1	2	2
40	1	2	2
41	1	2	2
42	1	2	2
43	2	1	2
44	1	2	2
45	1	2	2
46	1	2	2
47	1	2	2
48	1	2	2
49	1	2	2
50	1	2	2
51	1	2	2
52	1	2	2
53	1	2	2
54	2	1	2
55	1	2	2
56	1	2	2
57	1	2	2
58	1	2	2
59	1	2	2
60	1	2	2
61	1	2	2
62	1	2	2
63	2	1	2
64	1	2	2
65	1	2	2
66	1	2	2
67	1	2	2
68	1	2	2
69	1	2	2
70	1	2	2
71	1	2	1
72	1	2	2
73	1	2	2
74	1	2	2
75	1	2	2
76	1	2	2

77	2	1	2
78	1	2	2
79	1	2	2
80	1	2	2
81	1	2	2
82	1	2	2
83	2	1	2
84	1	2	2
85	2	1	2
86	1	2	2
87	1	2	2
88	1	1	2
89	1	2	2
90	1	2	2
91	1	2	2
92	1	2	2
93	1	2	2
94	1	2	2
95	1	2	2
96	1	1	2
97	1	2	2
98	1	2	2
99	1	2	2
100	1	2	2
101	1	2	2
102	1	2	2
103	1	2	2
104	1	2	2
105	1	2	2
106	1	2	2
107	1	2	2
108	1	2	2
109	1	2	2
110	1	2	2
111	1	2	2
112	miss	miss	1
113	1	2	1
114	1	2	1
115	1	2	2
116	1	2	1
117	1	2	2
118	1	2	2
119	1	2	2
120	2	1	2
121	1	2	2
122	2	1	2
123	1	2	2
124	1	2	2
125	1	2	2
126	1	2	2
127	1	2	2
128	1	2	2
129	1	2	2
130	1	2	2
131	1	2	2
132	1	2	2
133	1	2	2
134	1	2	2
135	1	2	2
136	2	1	2
137	1	2	2
138	1	2	2
139	1	2	2
140	1	2	2
141	2	1	2
142	1	2	2
143	2	1	2
144	1	2	2
145	1	2	2
146	2	1	2
147	1	2	1
148	1	2	1
149	2	1	1
150	1	2	2
151	2	1	2
152	1	2	1
153	1	2	2
154	1	2	2
155	1	2	2
156	1	2	2
157	1	2	2
158	1	2	2
159	1	2	2
160	1	2	2
161	1	2	2

EXCLI Journal 2021;20:444-453 – ISSN 1611-2156
Received: January 16, 2021, accepted: February 16, 2021, published: February 22, 2021

162	1	2	2
163	1	2	2
164	1	2	2
165	1	2	2
166	1	2	2
167	1	2	2
168	1	2	2
169	1	2	2
170	1	2	2
171	1	2	2
172	1	2	2
173	1	2	2
174	1	2	2
175	1	2	2
176	1	2	2
177	1	2	2
178	1	2	2
179	1	2	2
180	1	2	2
181	1	2	2
182	1	2	2
183	miss	miss	2
184	1	2	1
185	1	2	2
186	1	2	2
187	1	2	2
188	1	2	2
189	1	2	2
190	1	2	2
191	1	2	2
192	1	2	2
193	1	2	2
194	1	2	2
195	1	2	2
196	1	2	2
197	1	2	2
198	1	2	2
199	1	2	2
200	1	2	1
201	1	2	2
202	1	2	2
203	1	2	2
204	1	2	2
205	1	2	2
206	1	2	1
207	2	1	1
208	1	2	2
209	2	1	2
210	1	2	2
211	1	2	2
212	1	2	2
213	1	2	2
214	1	2	2
215	1	2	2
216	1	2	2
217	1	2	2
218	1	2	2
219	1	2	2
220	1	2	2
221	1	2	2
222	1	2	2
223	1	2	2
224	1	2	2
225	1	2	2
226	1	2	2
227	1	2	2
228	1	2	1
229	1	2	1
230	1	2	2
231	1	2	1
232	1	2	1
233	1	2	2
234	1	2	2
235	2	1	2
236	1	2	2
237	2	1	2
238	1	2	2
239	1	2	2
240	1	2	2
241	1	2	2
242	1	2	2
243	1	2	2
244	1	2	2
245	1	2	2
246	1	2	2

EXCLI Journal 2021;20:444-453 – ISSN 1611-2156
Received: January 16, 2021, accepted: February 16, 2021, published: February 22, 2021

247	1	2	2
248	1	2	1
249	1	2	1
250	1	2	1
251	1	2	1
252	1	2	1
253	1	2	1
254	1	2	1
255	1	2	1
256	1	2	1
257	1	2	1
258	1	2	2
259	1	2	2
260	1	2	2
261	1	2	2
262	1	2	2
263	1	2	2
264	1	2	2
265	1	2	2
266	1	2	2
267	1	2	2
268	1	2	2

EXCLI Journal 2021;20:444-453 – ISSN 1611-2156
Received: January 16, 2021, accepted: February 16, 2021, published: February 22, 2021

Supplementary Table 5: Raw data of in-hospital management of SARS-CoV-2 infected patients with or without diabetes which is shown in Table 2 of the manuscript (part 1).

ID	Group 1: Control 2: Case	MF 1: No 2: Yes	Glibenclamide 1: No 2: Yes	Gliclazide 1: No 2: Yes	Lantus 1: No 2: Yes	Pioglitazone 1: No 2: Yes	Insulin 1: No 2: Yes	Dyazide 1: No 2: Yes	Repaglinide 1: No 2: Yes	NovoRapid 1: No 2: Yes	Zipmet 1: No 2: Yes	Zinc Plus 1: No 2: Yes	CRO 1: No 2: Yes	HCQ 1: No 2: Yes	AZM 1: No 2: Yes
1	1	1	1	1	1	1	1	1	1	1	1	1	1	2	2
2	1	1	1	1	1	1	1	1	1	1	1	1	2	1	1
3	1	1	1	1	1	1	1	1	1	1	1	1	1	2	2
4	1	1	1	1	1	1	1	1	1	1	1	1	1	1	2
5	1	1	1	1	1	1	1	1	1	1	1	1	1	1	2
6	1	1	1	1	1	1	1	1	1	1	1	1	2	1	2
7	1	1	1	1	1	1	1	1	1	1	1	1	1	1	2
8	1	1	1	1	1	1	1	1	1	1	1	1	1	2	2
9	1	1	1	1	1	1	1	1	1	1	1	1	1	2	2
10	1	1	1	1	1	1	1	1	1	1	1	1	2	1	1
11	1	1	1	1	1	1	1	1	1	1	1	1	2	1	1
12	1	1	1	1	1	1	1	1	1	1	1	1	1	1	2
13	1	1	1	1	1	1	1	1	1	1	1	1	1	2	2
14	1	1	1	1	1	1	1	1	1	1	1	1	1	2	2
15	1	1	1	1	1	1	1	1	1	1	1	1	1	2	2
16	1	1	1	1	1	1	1	1	1	1	1	1	2	2	2
17	1	1	1	1	1	1	1	1	1	1	1	1	1	1	2
18	1	1	1	1	1	1	1	1	1	1	1	1	1	2	1
19	1	1	1	1	1	1	1	1	1	1	1	1	2	2	2
20	1	1	1	1	1	1	1	1	1	1	1	1	2	2	2
21	1	1	1	1	1	1	1	1	1	1	1	1	1	1	1
22	1	1	1	1	1	1	1	1	1	1	1	1	1	1	2
23	1	1	1	1	1	1	1	1	1	1	1	1	1	1	2
24	1	1	1	1	1	1	1	1	1	1	1	1	1	1	2
25	1	1	1	1	1	1	1	1	1	1	1	1	2	2	2
26	1	1	1	1	1	1	1	1	1	1	1	1	1	1	1
27	1	1	1	1	1	1	1	1	1	1	1	1	1	1	2
28	1	1	1	1	1	1	1	1	1	1	1	1	1	2	1
29	1	1	1	1	1	1	1	1	1	1	1	1	1	2	2
30	1	1	1	1	1	1	1	1	1	1	1	1	1	2	2
31	1	1	1	1	1	1	1	1	1	1	1	1	2	1	1
32	1	1	1	1	1	1	1	1	1	1	1	1	1	1	2
33	1	1	1	1	1	1	1	1	1	1	1	1	1	2	2
34	1	1	1	1	1	1	1	1	1	1	1	1	1	1	2
35	1	1	1	1	1	1	1	1	1	1	1	1	1	2	2
36	1	1	1	1	1	1	1	1	1	1	1	1	1	2	2
37	1	1	1	1	1	1	1	1	1	1	1	1	2	2	2
38	1	1	1	1	1	1	1	1	1	1	1	1	1	1	2
39	1	1	1	1	1	1	1	1	1	1	1	1	1	1	1
40	1	1	1	1	1	1	1	1	1	1	1	1	1	1	2
41	1	1	1	1	1	1	1	1	1	1	1	1	1	2	1
42	1	1	1	1	1	1	1	1	1	1	1	1	1	1	2
43	1	1	1	1	1	1	1	1	1	1	1	1	1	1	1
44	1	1	1	1	1	1	1	1	1	1	1	1	1	2	2
45	1	1	1	1	1	1	1	1	1	1	1	1	1	2	2
46	1	1	1	1	1	1	1	1	1	1	1	1	1	1	2

47	1	1	1	1	1	1	1	1	1	1	1	1	1	1	2
48	1	1	1	1	1	1	1	1	1	1	1	1	2	2	1
49	1	1	1	1	1	1	1	1	1	1	1	1	1	2	2
50	1	1	1	1	1	1	1	1	1	1	1	1	2	1	1
51	1	1	1	1	1	1	1	1	1	1	1	1	1	2	1
52	1	1	1	1	1	1	1	1	1	1	1	1	2	2	1
53	1	1	1	1	1	1	1	1	1	1	1	1	1	2	2
54	1	1	1	1	1	1	1	1	1	1	1	1	1	2	1
55	1	1	1	1	1	1	1	1	1	1	1	1	1	1	2
56	1	1	1	1	1	1	1	1	1	1	1	1	1	2	2
57	1	1	1	1	1	1	1	1	1	1	1	1	1	1	1
58	1	1	1	1	1	1	1	1	1	1	1	1	1	1	2
59	1	1	1	1	1	1	1	1	1	1	1	1	2	1	2
60	1	1	1	1	1	1	1	1	1	1	1	1	1	1	1
61	1	1	1	1	1	1	1	1	1	1	1	1	2	1	2
62	1	1	1	1	1	1	1	1	1	1	1	1	1	2	2
63	1	1	1	1	1	1	1	1	1	1	1	1	1	1	2
64	1	1	1	1	1	1	1	1	1	1	1	1	2	1	2
65	1	1	1	1	1	1	1	1	1	1	1	1	1	2	2
66	1	1	1	1	1	1	1	1	1	1	1	1	2	1	2
67	1	1	1	1	1	1	1	1	1	1	1	1	1	2	2
68	1	1	1	1	1	1	1	1	1	1	1	1	1	1	2
69	1	1	1	1	1	1	1	1	1	1	1	1	1	2	2
70	1	1	1	1	1	1	1	1	1	1	1	1	1	1	2
71	1	1	1	1	1	1	1	1	1	1	1	1	1	1	2
72	1	1	1	1	1	1	1	1	1	1	1	1	2	1	2
73	1	1	1	1	1	1	1	1	1	1	1	1	1	1	2
74	1	1	1	1	1	1	1	1	1	1	1	1	1	2	2
75	1	1	1	1	1	1	1	1	1	1	1	1	1	1	2
76	1	1	1	1	1	1	1	1	1	1	1	1	2	1	2
77	1	1	1	1	1	1	1	1	1	1	1	1	1	2	2
78	1	1	1	1	1	1	1	1	1	1	1	1	1	1	2
79	1	1	1	1	1	1	1	1	1	1	1	1	1	1	2
80	1	1	1	1	1	1	1	1	1	1	1	1	2	2	1
81	1	1	1	1	1	1	1	1	1	1	1	1	1	2	2
82	1	1	1	1	1	1	1	1	1	1	1	1	1	1	2
83	1	1	1	1	1	1	1	1	1	1	1	1	2	2	2
84	1	1	1	1	1	1	1	1	1	1	1	1	1	1	2
85	1	1	1	1	1	1	1	1	1	1	1	1	1	2	2
86	1	1	1	1	1	1	1	1	1	1	1	1	2	1	2
87	1	1	1	1	1	1	1	1	1	1	1	1	1	2	2
88	1	1	1	1	1	1	1	1	1	1	1	1	1	2	2
89	1	1	1	1	1	1	1	1	1	1	1	1	1	1	2
90	1	1	1	1	1	1	1	1	1	1	1	1	1	2	2
91	1	1	1	1	1	1	1	1	1	1	1	1	2	2	2
92	1	1	1	1	1	1	1	1	1	1	1	1	1	1	2
93	1	1	1	1	1	1	1	1	1	1	1	1	1	2	2
94	1	1	1	1	1	1	1	1	1	1	1	1	1	2	2
95	1	1	1	1	1	1	1	1	1	1	1	1	1	1	2
96	1	1	1	1	1	1	1	1	1	1	1	1	1	2	1
97	1	1	1	1	1	1	1	1	1	1	1	1	1	2	1
98	1	1	1	1	1	1	1	1	1	1	1	1	2	2	2
99	1	1	1	1	1	1	1	1	1	1	1	1	1	2	1
100	1	1	1	1	1	1	1	1	1	1	1	1	1	1	2
101	1	1	1	1	1	1	1	1	1	1	1	1	1	2	2
102	1	1	1	1	1	1	1	1	1	1	1	1	1	1	2

EXCLI Journal 2021;20:444-453 – ISSN 1611-2156
Received: January 16, 2021, accepted: February 16, 2021, published: February 22, 2021

103	1	1	1	1	1	1	1	1	1	1	1	1	1	1	2
104	1	1	1	1	1	1	1	1	1	1	1	1	1	1	2
105	1	1	1	1	1	1	1	1	1	1	1	1	1	1	2
106	1	1	1	1	1	1	1	1	1	1	1	1	1	1	2
107	1	1	1	1	1	1	1	1	1	1	1	1	1	2	2
108	1	1	1	1	1	1	1	1	1	1	1	1	1	2	2
109	1	1	1	1	1	1	1	1	1	1	1	1	2	2	2
110	1	1	1	1	1	1	1	1	1	1	1	1	1	2	2
111	1	1	1	1	1	1	1	1	1	1	1	1	1	2	2
112	1	1	1	1	1	1	1	1	1	1	1	1	1	2	2
113	1	1	1	1	1	1	1	1	1	1	1	1	1	2	1
114	1	1	1	1	1	1	1	1	1	1	1	1	1	2	2
115	1	1	1	1	1	1	1	1	1	1	1	1	2	2	2
116	1	1	1	1	1	1	1	1	1	1	1	1	1	1	2
117	1	1	1	1	1	1	1	1	1	1	1	1	2	2	2
118	1	1	1	1	1	1	1	1	1	1	1	1	1	2	2
119	1	1	1	1	1	1	1	1	1	1	1	1	1	2	2
120	1	1	1	1	1	1	1	1	1	1	1	1	2	2	1
121	1	1	1	1	1	1	1	1	1	1	1	1	1	2	2
122	1	1	1	1	1	1	1	1	1	1	1	1	1	1	2
123	1	1	1	1	1	1	1	1	1	1	1	1	1	2	2
124	1	1	1	1	1	1	1	1	1	1	1	1	1	2	1
125	1	1	1	1	1	1	1	1	1	1	1	1	1	1	1
126	1	1	1	1	1	1	1	1	1	1	1	1	1	1	2
127	1	1	1	1	1	1	1	1	1	1	1	1	2	2	2
128	1	1	1	1	1	1	1	1	1	1	1	1	2	2	2
129	1	1	1	1	1	1	1	1	1	1	1	1	2	2	2
130	1	1	1	1	1	1	1	1	1	1	1	1	2	2	1
131	1	1	1	1	1	1	1	1	1	1	1	1	1	2	2
132	1	1	1	1	1	1	1	1	1	1	1	1	1	2	2
133	1	1	1	1	1	1	1	1	1	1	1	1	2	2	2
134	1	1	1	1	1	1	1	1	1	1	1	1	1	2	1
135	1	1	1	1	1	1	1	1	1	1	1	1	1	2	2
136	1	1	1	1	1	1	1	1	1	1	1	1	1	2	2
137	1	1	1	1	1	1	1	1	1	1	1	1	2	2	2
138	1	1	1	1	1	1	1	1	1	1	1	1	2	2	2
139	1	1	1	1	1	1	1	1	1	1	1	1	2	2	2
140	1	1	1	1	1	1	1	1	1	1	1	1	1	2	2
141	1	1	1	1	1	1	1	1	1	1	1	1	1	2	2
142	2	2	1	1	1	1	1	1	1	1	1	1	2	2	2
143	2	2	1	1	1	1	2	1	1	1	1	1	2	2	2
144	2	2	1	1	1	1	1	1	1	1	1	1	2	2	1
145	2	2	1	1	1	1	1	1	1	1	1	1	1	2	2
146	2	2	1	1	1	1	2	1	1	1	1	1	1	1	2
147	2	1	1	1	1	1	2	1	1	1	1	1	1	2	1
148	2	2	1	1	1	1	2	1	1	1	1	1	2	2	2
149	2	2	1	1	1	1	2	1	1	1	1	1	1	1	1
150	2	2	1	1	1	1	1	1	1	1	1	1	1	1	2
151	2	1	1	1	1	1	2	1	1	1	1	1	2	2	1
152	2	2	2	1	1	1	2	1	1	1	1	1	1	1	1
153	2	2	1	1	1	1	2	1	1	1	1	1	1	1	2
154	2	1	1	1	1	1	2	1	1	1	1	1	1	2	1
155	2	2	1	1	1	1	1	1	1	1	1	1	1	1	1
156	2	1	1	1	1	1	2	1	1	1	1	1	1	1	2
157	2	1	1	1	1	1	2	1	1	1	1	1	2	1	1
158	2	1	1	1	1	1	2	1	1	1	1	1	1	1	1

EXCLI Journal 2021;20:444-453 – ISSN 1611-2156
Received: January 16, 2021, accepted: February 16, 2021, published: February 22, 2021

159	2	2	2	1	1	1	2	1	1	1	1	1	1	1	2
160	2	2	2	1	1	1	2	1	1	1	1	1	1	1	2
161	2	2	2	1	1	1	2	1	1	1	1	1	1	1	1
162	2	2	1	1	1	1	1	2	1	1	1	1	1	2	2
163	2	1	1	1	1	1	1	1	1	1	1	1	1	1	2
164	2	2	1	1	1	1	1	1	1	1	1	1	2	2	1
165	2	1	1	1	1	1	2	1	1	1	1	1	1	2	1
166	2	2	1	1	1	1	1	1	1	1	1	1	1	1	2
167	2	1	1	1	1	1	2	1	1	1	1	1	1	1	2
168	2	2	1	1	1	1	2	2	1	1	1	1	1	1	1
169	2	2	1	1	1	1	1	1	1	1	1	1	1	2	2
170	2	1	1	1	1	1	2	1	1	1	1	1	1	2	1
171	2	1	2	1	1	1	1	1	1	1	1	1	1	2	1
172	2	1	1	1	1	1	1	1	1	1	1	1	1	1	2
173	2	1	1	1	2	1	2	1	1	2	1	1	1	1	2
174	2	2	1	1	1	1	2	1	1	1	1	1	2	2	2
175	2	2	1	1	1	1	1	1	1	1	1	1	1	1	2
176	2	2	2	1	1	1	1	1	1	1	1	1	1	1	2
177	2	1	1	1	1	1	1	1	1	1	1	1	1	1	2
178	2	1	1	2	1	1	1	1	1	1	1	1	2	2	2
179	2	2	1	1	1	1	2	1	1	1	1	1	1	1	2
180	2	2	1	1	1	1	1	1	1	1	1	1	1	2	2
181	2	2	1	1	1	1	2	1	1	1	1	1	1	2	1
182	2	2	1	1	1	1	2	1	1	1	1	1	1	2	1
183	2	2	1	1	1	1	1	1	1	1	1	1	2	1	2
184	2	1	1	1	1	1	2	1	1	1	1	1	2	2	1
185	2	2	1	1	1	1	1	1	1	1	1	1	1	2	2
186	2	1	1	1	1	1	2	1	1	1	1	1	1	1	2
187	2	2	2	1	1	1	1	1	1	1	1	1	1	1	2
188	2	2	1	1	1	1	1	1	1	1	1	1	1	2	2
189	2	2	1	1	1	1	2	1	1	1	1	1	2	2	2
190	2	2	1	1	1	1	1	1	1	1	1	1	2	2	2
191	2	1	1	1	1	1	1	1	1	1	1	1	1	2	2
192	2	2	2	1	1	1	1	1	1	1	1	1	2	2	2
193	2	2	1	1	1	1	1	1	1	1	1	1	1	1	2
194	2	1	1	1	1	1	1	1	1	1	1	1	1	1	2
195	2	2	1	1	1	1	1	1	1	1	1	1	2	2	1
196	2	1	2	1	1	1	2	1	1	1	1	1	1	1	1
197	2	1	2	1	2	1	1	1	1	1	1	1	2	2	2
198	2	2	1	1	1	1	1	1	1	1	1	1	1	2	2
199	2	2	1	1	1	1	1	1	1	1	1	1	2	2	2
200	2	1	1	1	1	1	1	1	1	1	1	1	1	1	1
201	2	1	2	1	1	1	2	1	1	1	1	1	1	2	2
202	2	2	1	2	1	2	1	1	1	1	1	1	1	1	2
203	2	2	1	1	1	1	2	1	1	1	1	1	1	2	2
204	2	1	1	1	1	1	2	1	1	1	1	1	1	2	2
205	2	2	1	1	1	1	1	1	1	1	1	1	2	2	2
206	2	1	1	1	1	1	2	1	1	1	1	1	1	2	2
207	2	1	1	1	1	1	2	1	1	1	1	1	1	2	1
208	2	2	1	2	1	1	1	1	1	1	1	1	2	2	2
209	2	2	1	2	1	1	1	1	1	1	1	1	2	2	2
210	2	2	1	1	1	1	2	1	1	1	1	1	1	1	2
211	2	1	1	2	1	1	2	1	1	1	1	1	2	2	2
212	2	2	1	1	1	1	2	1	1	1	1	1	2	2	2
213	2	1	1	2	1	1	2	2	1	1	1	1	1	1	1
214	2	1	1	1	1	1	2	1	1	1	1	1	1	2	2

EXCLI Journal 2021;20:444-453 – ISSN 1611-2156
Received: January 16, 2021, accepted: February 16, 2021, published: February 22, 2021

215	2	2	1	1	1	1	1	1	1	1	1	1	1	1	1
216	2	1	1	1	1	1	2	1	1	1	1	1	1	2	1
217	2	1	1	1	1	1	2	1	1	1	1	1	1	2	2
218	2	2	1	1	1	1	2	1	1	1	1	1	1	2	1
219	2	1	1	1	1	1	2	1	1	1	1	1	1	2	2
220	2	1	1	1	1	1	2	1	1	2	1	1	1	1	1
221	2	2	1	1	1	1	1	1	1	1	1	1	1	2	2
222	2	2	2	1	1	1	2	1	1	1	1	1	1	2	1
223	2	1	1	1	1	1	2	1	1	1	1	1	1	1	2
224	2	2	2	1	1	1	2	1	1	1	1	1	1	2	2
225	2	1	1	1	1	1	2	1	1	1	1	1	1	1	2
226	2	1	1	1	1	1	1	1	1	1	1	1	1	1	1
227	2	1	1	1	1	1	2	1	1	1	1	1	1	2	1
228	2	2	1	1	1	1	1	1	1	1	1	1	1	1	2
229	2	1	1	1	1	1	2	1	1	1	1	1	1	2	2
230	2	2	1	1	1	1	2	1	1	1	1	1	1	2	2
231	2	1	1	1	1	1	2	1	1	1	1	1	1	2	1
232	2	2	1	1	1	1	1	1	1	1	1	1	1	2	1
233	2	1	1	1	1	1	2	1	1	1	1	1	1	2	2
234	2	1	1	1	1	1	2	1	1	1	1	1	1	2	2
235	2	1	1	1	1	1	2	1	1	1	1	1	1	2	2
236	2	1	1	1	1	1	2	1	1	1	1	1	1	2	2
237	2	1	2	1	1	1	2	1	2	1	1	1	1	2	2
238	2	1	1	1	1	1	2	1	1	1	1	1	1	2	2
239	2	2	1	1	1	1	1	1	1	1	1	1	1	2	2
240	2	1	2	1	1	1	2	2	1	1	1	1	1	1	1
241	2	1	1	1	1	1	2	1	1	1	1	1	1	2	2
242	2	2	1	1	1	1	1	1	1	1	1	1	1	2	2
243	2	1	1	1	1	1	2	1	1	1	1	1	1	2	2
244	2	1	1	1	1	1	1	1	2	1	1	2	1	2	2
245	2	2	1	2	1	1	2	1	1	1	1	1	1	1	1
246	2	1	2	1	1	1	1	1	1	1	1	1	1	1	1
247	2	1	1	1	1	1	2	1	2	1	1	2	1	2	1
248	2	2	1	1	1	1	1	1	1	1	1	1	1	2	2
249	2	1	1	1	1	1	2	1	1	1	1	1	1	2	1
250	2	2	1	1	1	1	1	1	1	1	1	1	1	2	1
251	2	1	2	1	1	1	1	1	1	1	1	1	1	2	2
252	2	2	2	1	1	1	1	1	1	1	1	1	1	2	2
253	2	2	1	1	1	1	2	1	1	1	1	1	1	1	1
254	2	2	1	1	1	1	1	1	1	1	1	1	1	2	2
255	2	2	1	1	1	1	1	1	1	1	1	1	1	2	2
256	2	2	2	1	1	1	2	1	1	1	1	1	1	2	1
257	2	1	1	1	1	1	2	1	1	1	1	1	1	1	1
258	2	2	1	1	1	1	2	1	1	1	1	1	1	2	2
259	2	1	2	1	1	1	2	1	1	1	1	1	1	2	2
260	2	1	1	1	1	1	2	1	1	1	1	1	2	1	2
261	2	2	1	1	1	1	1	1	1	1	1	1	1	2	2
262	2	2	1	1	1	1	1	1	1	1	1	1	1	2	1
263	2	2	1	1	1	1	2	1	1	1	1	1	1	2	1
264	2	1	1	2	1	1	1	1	1	1	1	1	1	2	2
265	2	2	2	1	1	1	1	1	1	1	1	1	1	2	2
266	2	1	2	1	1	1	1	1	1	1	1	1	1	2	1
267	2	2	2	1	1	1	1	1	1	1	1	1	1	2	1
268	2	1	2	1	1	1	2	1	1	1	2	1	1	1	2

ID: Identity number, MF: Metformin, CRO: ceftriaxone, HCQ: Hydroxychloroquine, AZM: Azithromycin

Supplementary Table 6: Raw data of in-hospital management of SARS-CoV-2 infected patients with or without diabetes which is shown in Table 2 of the manuscript (part 2).

ID	Naproxen 1: No 2: Yes	Enoxa 1: No 2: Yes	IVIg 1: No 2: Yes	ReciGen 1: No 2: Yes	AT 1: No 2: Yes	VAN 1: No 2: Yes	APAP 1: No 2: Yes	difen 1: No 2: Yes	Panta 1: No 2: Yes	MEM 1: No 2: Yes	VIT C 1: No 2: Yes	Airokast 1: No 2: Yes	Lasix 1: No 2: Yes	PB 1: No 2: Yes	Digoxin 1: No 2: Yes	Cefepime 1: No 2: Yes
1	1	1	1	1	1	1	1	1	1	1	1	1	1	1	1	1
2	1	2	1	1	1	1	2	2	1	1	1	1	2	2	2	1
3	2	1	1	1	1	1	1	1	1	1	1	1	1	1	1	2
4	2	2	1	1	1	1	1	1	2	1	1	1	1	1	1	1
5	2	1	1	2	1	1	1	2	1	1	1	1	1	1	1	1
6	2	1	1	1	1	1	1	2	2	1	1	1	1	1	1	1
7	1	2	2	2	1	1	1	2	1	1	1	1	1	1	1	1
8	2	1	1	2	1	1	1	1	1	1	1	1	1	1	1	1
9	1	1	1	1	1	1	1	2	1	1	1	1	1	1	1	1
10	2	1	1	1	1	1	1	1	1	1	1	1	1	1	1	1
11	1	1	1	1	1	1	2	1	1	1	1	1	1	1	1	1
12	2	1	1	1	2	1	1	1	1	1	1	1	1	1	1	1
13	1	1	1	1	1	1	1	1	1	1	1	1	1	1	1	1
14	1	1	1	1	1	1	1	1	1	1	1	1	1	1	1	1
15	2	1	1	1	1	1	1	1	1	1	1	1	1	1	1	1
16	2	2	1	1	2	2	2	1	1	1	1	1	1	1	1	1
17	1	2	1	1	1	1	1	1	1	1	1	1	1	1	1	1
18	2	1	1	1	2	1	1	1	1	1	1	1	1	1	1	1
19	1	2	1	1	1	1	1	1	1	1	1	1	1	1	1	1
20	2	2	1	1	1	1	1	2	1	1	2	1	1	1	1	1
21	2	2	1	1	2	1	2	2	1	1	1	1	1	1	1	1
22	2	2	1	1	2	1	1	1	1	1	1	1	1	1	1	1
23	1	2	1	1	1	1	1	1	1	1	1	1	1	1	1	1
24	2	2	2	1	1	1	1	1	1	1	1	1	1	1	1	2
25	2	1	1	1	1	1	1	1	1	1	1	1	1	1	1	1
26	2	2	1	1	1	1	1	1	1	1	1	1	1	1	1	1
27	2	2	1	1	1	1	1	1	1	1	1	1	1	1	1	1
28	1	2	1	1	1	1	2	1	1	2	1	1	1	1	1	2
29	2	1	1	1	1	1	1	1	1	1	1	1	1	1	1	1
30	2	2	1	1	1	1	1	1	1	1	1	1	1	1	1	1
31	1	1	1	1	1	1	1	1	1	1	1	1	1	1	1	1
32	2	2	1	1	2	1	1	1	2	1	1	1	1	1	1	1
33	2	2	1	1	1	1	1	1	1	1	1	1	1	1	1	1
34	2	2	1	1	2	1	1	2	1	1	1	1	1	1	1	1
35	1	1	1	1	2	1	1	1	1	1	1	1	1	1	1	1
36	2	1	1	1	2	1	1	2	2	1	1	1	1	1	1	1
37	1	2	1	1	1	1	1	2	1	1	1	1	1	1	1	1
38	1	1	1	1	1	1	2	1	1	1	1	1	1	1	1	1
39	2	1	1	1	2	1	1	1	1	1	1	1	1	1	1	1
40	1	2	1	1	2	1	1	1	1	1	1	2	1	1	1	1
41	2	1	1	1	1	1	1	1	1	1	1	1	1	1	1	1
42	2	2	1	1	2	1	1	1	1	1	1	1	1	1	1	1
43	2	2	1	1	1	1	1	1	1	1	1	1	1	1	1	1
44	2	2	1	1	1	1	1	2	1	1	1	1	2	1	1	1
45	1	1	1	1	1	1	1	1	1	1	1	1	1	1	1	1
46	2	1	1	1	1	1	1	1	1	1	1	1	1	1	1	1

EXCLI Journal 2021;20:444-453 – ISSN 1611-2156
Received: January 16, 2021, accepted: February 16, 2021, published: February 22, 2021

47	2	2	1	1	2	1	1	2	1	1	1	1	2	1	1	1
48	2	2	1	1	1	1	1	2	1	1	1	1	1	1	1	1
49	2	1	1	1	2	1	1	2	1	1	1	1	1	1	1	1
50	1	2	1	1	1	1	2	1	1	1	1	1	1	1	1	1
51	2	1	1	1	1	1	1	1	1	1	1	1	1	1	1	1
52	1	1	1	1	1	1	1	1	1	1	1	1	1	1	1	1
53	1	1	1	1	1	1	1	1	1	1	1	1	1	1	1	1
54	1	1	1	1	1	1	1	1	1	1	1	1	1	1	1	1
55	2	1	1	1	1	1	1	1	1	1	1	1	1	1	1	1
56	2	1	1	1	1	1	1	1	1	1	1	1	1	1	1	1
57	2	1	1	1	1	1	2	2	1	1	1	1	1	1	1	1
58	1	2	1	1	1	1	1	2	2	1	1	1	1	1	1	1
59	2	1	2	2	1	1	1	1	1	1	1	1	1	1	1	1
60	2	2	1	1	1	1	1	1	1	1	1	1	1	1	1	1
61	1	1	1	1	1	1	1	1	1	1	1	1	1	1	1	1
62	1	1	1	2	1	1	2	1	1	1	1	1	1	1	1	1
63	2	1	1	1	1	1	1	1	1	1	1	1	1	1	1	1
64	2	1	1	1	1	1	1	1	1	1	1	1	1	1	1	1
65	1	1	1	2	1	1	1	1	1	1	1	1	1	1	1	1
66	2	1	2	1	1	1	1	1	1	1	1	1	1	1	1	1
67	1	1	1	1	1	1	1	1	1	1	1	1	1	1	1	1
68	1	1	1	1	1	1	1	1	1	1	1	1	1	1	1	1
69	2	1	1	1	1	1	1	1	1	1	1	1	1	1	1	1
70	2	1	1	2	1	1	1	1	1	1	1	1	1	1	1	1
71	2	2	1	1	1	1	1	1	2	1	1	1	1	1	1	1
72	1	1	1	2	1	1	1	1	1	1	1	1	1	1	1	1
73	1	2	1	1	1	1	1	2	1	1	1	1	1	1	1	1
74	2	1	1	1	1	1	1	1	1	1	1	1	1	1	1	1
75	2	2	1	1	1	1	1	1	1	1	1	1	1	1	1	1
76	2	1	1	1	1	1	1	1	1	1	1	1	1	1	1	1
77	2	1	1	1	1	1	1	1	1	1	1	1	1	1	1	1
78	1	1	1	1	1	1	2	2	1	1	1	1	1	1	1	1
79	1	2	1	1	1	1	1	1	1	1	1	1	1	1	1	1
80	1	1	1	1	1	1	1	1	1	1	1	1	1	1	1	1
81	2	2	1	1	1	1	1	1	1	1	1	1	1	1	1	1
82	2	1	1	1	1	1	1	1	1	1	1	1	1	1	1	1
83	1	1	1	1	1	1	1	1	1	1	1	1	1	1	1	1
84	2	1	1	1	1	1	1	1	1	1	1	1	1	1	1	1
85	2	1	1	1	1	1	1	1	1	1	1	1	1	1	1	1
86	2	1	1	1	1	1	1	1	1	1	1	1	1	1	1	1
87	2	1	1	1	1	1	1	2	1	1	1	1	1	1	1	1
88	2	2	1	1	1	1	2	2	1	1	1	1	1	1	1	1
89	2	2	1	1	2	1	1	1	2	1	1	1	1	1	1	1
90	2	2	1	1	2	1	1	1	1	1	1	1	1	1	1	1
91	1	2	1	1	1	1	1	1	2	1	1	1	1	1	1	1
92	1	1	1	1	2	1	1	1	1	1	1	1	1	1	1	1
93	2	1	1	1	1	1	1	2	1	1	1	1	1	1	1	1
94	1	1	1	1	2	1	2	1	2	1	1	1	1	1	1	1
95	2	1	1	1	1	1	1	1	1	1	1	1	1	1	1	1
96	2	2	1	1	1	1	1	2	1	1	1	1	1	1	1	1
97	2	1	1	1	2	1	2	2	1	1	1	1	1	1	1	1
98	1	1	1	1	1	1	1	2	1	1	1	1	1	1	1	1
99	1	1	1	1	1	1	1	1	1	1	1	1	1	1	1	1
100	2	1	1	1	1	1	1	1	2	1	1	1	1	1	1	1
101	2	2	1	1	1	1	1	1	1	1	2	1	1	1	1	1
102	2	1	1	1	1	1	1	1	1	1	1	1	1	1	1	1

EXCLI Journal 2021;20:444-453 – ISSN 1611-2156
Received: January 16, 2021, accepted: February 16, 2021, published: February 22, 2021

103	2	1	1	1	1	1	1	1	1	1	1	1	1	1	1	1
104	2	2	1	1	2	1	1	1	1	1	1	1	1	1	1	1
105	2	2	1	1	2	1	1	1	1	1	1	1	1	1	1	1
106	2	1	1	1	1	1	1	1	1	1	1	1	1	1	1	1
107	2	1	1	1	1	1	1	1	1	1	1	1	1	1	1	1
108	2	1	1	1	1	1	1	1	1	1	1	1	1	1	1	1
109	2	1	1	1	1	1	1	1	1	1	1	1	1	1	1	1
110	2	1	1	1	1	1	1	1	1	1	1	1	1	1	1	1
111	2	1	1	1	1	1	1	1	1	1	1	1	1	1	1	1
112	1	1	1	1	1	1	1	1	1	1	1	1	1	1	1	1
113	2	1	1	1	1	1	1	1	1	1	1	1	1	1	1	1
114	2	1	1	1	1	1	1	1	1	1	1	1	1	1	1	1
115	2	1	1	1	1	1	1	1	1	1	1	1	1	1	1	1
116	1	1	1	1	1	1	1	1	1	1	1	1	1	1	1	1
117	2	1	1	1	1	1	1	1	1	1	1	1	1	1	1	1
118	2	1	1	1	1	1	1	1	1	1	1	1	1	1	1	1
119	2	1	1	1	1	1	1	1	1	1	1	1	1	1	1	1
120	2	1	1	1	1	1	1	1	1	1	1	1	1	1	1	1
121	2	1	1	1	1	1	1	1	1	1	1	1	1	1	1	1
122	2	1	1	1	1	1	1	1	1	1	1	1	1	1	1	1
123	2	1	1	1	1	1	2	1	1	1	1	1	1	1	1	1
124	2	1	1	1	1	1	1	1	1	1	1	1	1	1	1	1
125	1	1	1	1	1	1	1	1	1	1	1	1	1	1	1	1
126	2	1	1	1	1	1	1	1	1	1	1	1	1	1	1	1
127	1	1	1	1	1	1	1	1	1	1	1	1	1	1	1	1
128	2	1	1	1	1	1	1	1	1	1	1	1	1	1	1	1
129	2	1	1	1	1	1	1	1	1	1	1	1	1	1	1	1
130	2	1	1	1	1	1	1	1	1	1	1	1	1	1	1	1
131	1	1	1	1	1	1	1	1	1	1	1	1	1	1	1	1
132	2	1	1	1	1	1	1	1	1	1	1	1	1	1	1	1
133	2	1	1	1	1	1	1	1	1	1	1	1	1	1	1	1
134	1	1	1	1	1	1	1	1	1	1	1	1	1	1	1	1
135	2	1	1	1	1	1	1	1	1	1	1	1	1	1	1	1
136	2	1	1	1	1	1	1	1	1	1	1	1	1	1	1	1
137	2	1	1	1	1	1	1	1	1	1	1	1	1	1	1	1
138	2	1	1	1	1	1	1	1	1	1	1	1	1	1	1	1
139	2	1	1	1	2	1	1	1	1	1	1	1	1	1	1	1
140	2	1	1	1	1	1	1	1	1	1	1	1	1	1	1	1
141	2	1	1	1	1	1	1	1	1	1	1	1	1	1	1	1
142	2	1	1	1	1	1	1	1	1	1	1	1	1	1	1	1
143	2	2	2	2	1	1	1	1	1	1	1	1	1	1	1	1
144	2	2	1	1	1	1	1	1	1	1	1	1	1	1	1	1
145	1	1	1	1	2	1	1	1	1	1	1	1	1	1	1	1
146	2	2	1	1	1	1	1	1	1	1	1	1	1	1	1	1
147	2	2	2	2	1	1	1	1	1	1	1	1	1	1	1	1
148	1	1	1	2	1	2	1	1	1	1	1	1	1	1	1	1
149	2	1	1	1	1	1	2	1	1	1	1	1	1	1	1	1
150	2	1	1	1	1	1	1	1	1	1	1	1	1	1	1	1
151	2	2	1	1	1	1	2	1	1	1	1	1	1	1	1	1
152	1	2	2	1	1	1	1	1	1	1	1	1	1	1	1	1
153	1	2	1	1	1	1	1	1	1	1	1	1	1	1	1	1
154	2	1	1	1	1	1	2	1	1	1	1	1	1	1	1	1
155	2	1	1	1	1	1	1	1	1	1	1	1	1	1	1	1
156	2	1	1	1	1	1	1	1	1	1	1	1	1	1	1	1
157	2	1	1	1	1	1	1	1	1	1	1	1	1	1	1	1
158	1	2	1	1	2	1	2	1	1	1	1	1	1	1	1	1

EXCLI Journal 2021;20:444-453 – ISSN 1611-2156
Received: January 16, 2021, accepted: February 16, 2021, published: February 22, 2021

159	1	1	1	1	1	1	1	2	2	1	1	1	1	1	1	1
160	1	2	1	1	1	1	1	2	1	1	1	1	1	1	1	1
161	2	2	2	1	2	1	1	1	1	1	1	1	1	1	1	1
162	2	1	1	1	1	1	1	1	1	1	1	1	1	1	1	1
163	2	1	1	1	1	1	2	1	1	1	1	1	1	1	1	1
164	2	1	1	1	1	1	1	1	1	1	1	1	1	1	1	1
165	1	1	1	1	1	2	1	1	1	2	1	1	1	1	1	1
166	2	1	1	1	1	1	1	1	1	1	1	1	1	1	1	1
167	1	1	1	1	1	1	1	1	1	1	1	1	1	1	1	1
168	2	1	1	1	1	1	1	1	2	1	1	1	1	1	1	1
169	2	1	1	1	1	1	1	1	2	1	1	1	1	1	1	1
170	1	2	2	1	1	1	1	1	1	1	1	1	1	1	1	1
171	2	1	1	1	1	1	1	1	1	1	1	1	1	1	1	1
172	1	2	1	1	1	1	1	1	1	1	1	1	2	1	1	1
173	1	2	1	1	1	1	1	2	1	1	1	1	1	1	1	1
174	1	1	2	1	1	1	1	1	1	1	1	1	1	1	1	1
175	2	1	1	2	1	1	1	1	1	1	1	1	1	1	1	1
176	2	2	1	1	1	1	1	1	1	1	2	1	1	1	1	1
177	2	1	1	1	1	1	1	2	1	1	1	1	1	1	1	1
178	1	2	1	1	2	1	1	1	1	1	1	1	1	1	1	1
179	1	2	1	1	1	1	1	1	1	1	1	1	1	1	1	1
180	2	2	1	1	1	1	2	1	1	1	1	1	1	1	1	1
181	2	1	1	1	2	1	1	1	1	1	1	1	1	1	1	2
182	2	1	1	1	1	1	1	1	1	1	1	1	1	1	1	1
183	1	1	1	1	1	1	1	1	1	1	1	1	1	1	1	1
184	2	1	1	1	1	1	1	1	1	1	1	1	1	1	1	1
185	1	2	1	1	1	1	1	1	1	1	1	1	1	1	1	1
186	1	1	1	1	2	1	1	1	1	1	1	1	1	1	1	1
187	1	1	1	1	1	1	1	1	1	1	1	1	1	1	1	1
188	2	2	1	1	2	1	1	1	2	1	1	1	1	1	1	1
189	1	2	1	1	1	1	1	1	1	1	1	1	1	1	1	1
190	2	1	1	1	1	1	1	1	1	1	1	1	1	1	1	1
191	2	1	1	1	1	1	1	1	1	1	1	1	2	1	1	1
192	2	1	1	1	1	1	1	1	1	1	1	1	1	1	1	1
193	2	1	1	1	1	1	1	1	1	1	1	1	1	1	1	1
194	1	1	1	1	1	1	1	2	1	1	1	1	1	1	1	1
195	2	2	1	1	1	1	1	1	1	1	1	1	1	1	1	1
196	2	2	2	2	1	1	1	1	1	1	1	1	1	1	1	1
197	1	1	1	1	2	1	2	2	1	1	1	1	1	1	1	1
198	2	1	1	1	1	1	1	1	1	1	1	1	1	1	1	1
199	2	1	1	1	1	1	1	1	1	1	1	1	1	1	1	1
200	1	1	1	1	1	1	1	1	1	1	1	1	1	1	1	1
201	2	1	1	1	1	1	1	1	1	1	1	1	1	1	1	1
202	2	2	1	1	1	1	1	1	1	1	1	1	1	1	1	1
203	1	1	1	1	1	1	1	1	1	1	1	1	1	1	1	1
204	1	2	1	1	1	1	1	1	1	1	1	1	1	1	1	1
205	2	1	1	1	1	1	1	1	1	1	1	1	1	1	1	1
206	2	1	1	1	1	1	1	1	1	1	1	1	1	1	1	2
207	1	1	1	1	1	1	2	1	1	1	1	1	1	1	1	1
208	1	1	1	1	1	1	1	1	1	1	1	1	1	1	1	1
209	2	2	1	1	1	1	1	1	1	1	1	1	1	1	1	1
210	1	2	1	1	1	1	1	1	1	1	1	1	1	1	1	1
211	1	2	1	2	1	1	1	1	1	1	1	1	1	1	1	1
212	1	1	2	2	1	1	1	1	1	1	1	1	1	1	1	1
213	2	2	1	2	1	1	1	1	1	1	1	1	1	1	1	1
214	1	1	1	1	1	1	1	1	1	1	1	1	1	1	1	1

EXCLI Journal 2021;20:444-453 – ISSN 1611-2156
Received: January 16, 2021, accepted: February 16, 2021, published: February 22, 2021

215	2	1	1	1	1	1	1	1	1	1	1	1	1	1	1	1
216	2	1	1	1	1	1	2	2	1	1	1	1	1	1	1	1
217	1	1	2	1	1	1	1	1	1	1	1	1	1	1	1	1
218	2	2	1	1	1	1	1	2	1	1	1	1	1	1	1	1
219	2	1	2	1	1	1	1	2	1	1	1	1	1	1	1	1
220	1	2	1	1	1	1	2	2	1	1	1	1	1	1	1	1
221	1	1	1	1	1	1	1	1	1	2	1	1	1	1	1	1
222	2	1	2	1	1	1	1	1	1	1	1	1	1	1	1	1
223	2	1	1	1	1	1	1	1	1	1	1	1	1	1	1	1
224	1	1	1	1	1	1	1	2	1	1	1	1	1	1	1	1
225	2	2	1	1	1	1	1	1	1	1	1	1	1	1	1	1
226	1	2	1	2	1	1	1	2	1	1	1	1	1	1	1	1
227	1	2	1	2	1	1	1	1	1	1	1	1	1	1	1	1
228	2	1	1	1	1	1	1	1	1	1	1	1	1	1	1	1
229	2	1	1	1	1	1	1	1	1	2	1	1	1	1	1	1
230	2	1	1	1	1	1	1	2	1	1	1	1	1	1	1	1
231	1	1	1	1	1	2	1	2	1	2	1	1	1	1	1	1
232	2	1	1	1	1	1	1	1	1	1	1	1	1	1	1	2
233	1	1	1	1	1	1	2	1	1	1	1	1	1	1	1	1
234	2	1	1	1	1	1	1	1	1	1	1	1	1	1	1	1
235	1	1	1	1	1	1	1	1	1	1	1	1	1	1	1	1
236	2	1	1	1	1	1	1	2	1	1	1	1	1	1	1	1
237	1	1	1	1	1	1	1	2	1	1	1	1	1	1	1	1
238	1	1	1	1	1	1	1	1	1	1	1	1	1	1	1	1
239	1	1	1	1	1	1	2	1	1	1	1	1	1	1	1	1
240	2	2	1	1	1	1	1	1	1	1	1	1	1	1	1	1
241	2	1	1	1	1	1	1	1	1	1	1	1	1	1	1	1
242	2	1	1	1	1	1	1	1	1	1	1	1	1	1	1	1
243	2	1	1	1	1	1	1	1	1	1	1	1	1	1	1	1
244	2	1	2	1	1	1	1	1	1	1	1	1	1	1	1	1
245	2	1	2	1	1	1	1	1	1	1	1	1	1	1	1	1
246	2	1	1	1	1	1	1	1	1	1	1	1	1	1	1	1
247	2	1	2	1	1	1	1	1	1	1	1	1	1	1	1	1
248	1	1	2	1	1	1	1	1	1	1	1	1	1	1	1	1
249	1	1	1	1	1	1	1	2	1	1	1	1	1	1	1	1
250	2	1	1	1	1	1	1	1	1	1	1	1	1	1	1	1
251	2	1	2	1	1	1	1	1	1	1	1	1	1	1	1	1
252	2	1	1	1	1	2	2	1	1	1	1	1	1	1	1	1
253	1	1	1	1	1	1	1	2	1	1	1	1	1	1	1	1
254	2	1	1	1	1	1	1	1	1	1	1	1	1	1	1	1
255	2	1	1	1	1	1	1	1	1	1	1	1	1	1	1	1
256	2	1	1	1	1	2	1	1	1	2	1	1	1	1	1	1
257	2	1	1	1	1	1	1	1	1	1	1	1	1	1	1	2
258	2	1	1	1	1	1	1	1	1	1	1	1	1	1	1	1
259	2	1	1	1	1	1	1	1	1	1	1	1	1	1	1	1
260	2	1	1	1	1	1	1	2	1	1	1	1	1	1	1	1
261	2	1	1	1	1	1	1	1	1	1	1	1	1	1	1	1
262	2	1	1	1	1	1	1	1	1	1	1	1	1	1	1	1
263	2	1	1	1	1	1	2	2	1	1	1	1	1	1	1	1
264	2	1	1	1	1	1	1	1	1	1	1	1	1	1	1	1
265	2	1	1	1	1	1	1	1	1	1	1	1	1	1	1	1
266	2	1	1	1	1	1	1	1	1	1	1	1	1	1	1	1
267	1	1	1	1	1	1	1	2	1	1	1	1	1	1	1	1
268	1	1	1	1	1	1	1	2	1	1	1	1	1	1	1	1

ID: Identity number, AT: Atorvastatin, VAN: Vancomycin, APAP: Acetaminophen, MEM: Meropenem, VIT C: Vitamin C, PB: Phenobarbital

EXCLI Journal 2021;20:444-453 – ISSN 1611-2156
Received: January 16, 2021, accepted: February 16, 2021, published: February 22, 2021

Supplementary Table 7: Raw data of in-hospital management of SARS-CoV-2 infected patients with or without diabetes which is shown in Table 2 of the manuscript (part 3).

ID	Losartan 1: No 2: Yes	Metoral 1: No 2: Yes	Nitrocontin 1: No 2: Yes	Apo-tel 1: No 2: Yes	pethi-dine 1: No 2: Yes	CAZ 1: No 2: Yes	Nephrovit 1: No 2: Yes	Heparin 1: No 2: Yes	Foradil 1: No 2: Yes	A.S.A 1: No 2: Yes	Captopril 1: No 2: Yes	Prometha-zine 1: No 2: Yes	Loperamid e 1: No 2: Yes	Methylpredniso-lone 1: No 2: Yes	Zinc sul-fate 1: No 2: Yes	Amlopres 1: No 2: Yes
1	1	1	1	1	1	1	1	1	1	1	1	1	1	1	1	1
2	1	1	1	1	1	1	1	1	1	1	1	1	1	1	1	1
3	1	1	1	1	1	1	1	1	1	1	1	1	1	1	1	1
4	2	1	1	1	1	1	1	1	1	1	1	1	1	1	1	1
5	1	1	1	1	1	1	1	1	1	1	1	1	1	1	1	1
6	1	1	1	1	1	1	1	1	1	1	1	1	1	1	1	1
7	1	1	1	1	1	1	1	1	1	1	1	1	1	1	1	1
8	1	1	1	1	1	1	1	1	1	1	1	1	1	1	1	1
9	1	1	1	1	1	1	1	1	1	1	1	1	1	1	1	1
10	1	2	2	1	1	1	1	1	1	1	1	1	1	1	1	1
11	1	1	1	2	2	1	1	1	1	1	1	1	1	1	1	1
12	1	1	1	1	1	1	1	1	1	1	1	1	1	1	1	1
13	1	1	1	1	1	1	1	1	1	1	1	1	1	1	1	1
14	1	1	1	1	1	1	1	1	1	1	1	1	1	1	1	1
15	1	1	1	1	1	1	1	1	1	1	1	1	1	1	1	1
16	1	1	1	1	1	1	1	1	1	1	1	1	1	1	1	1
17	1	1	1	1	1	1	1	1	1	1	1	1	1	1	1	1
18	1	1	1	1	1	1	1	1	1	1	1	1	1	1	1	1
19	1	1	1	1	1	1	1	1	1	1	1	1	1	1	1	1
20	1	1	1	1	1	1	1	1	1	1	1	1	1	1	1	1
21	1	1	1	2	1	1	1	1	1	1	1	1	1	1	1	1
22	1	1	1	1	1	1	1	1	1	1	1	1	1	1	1	1
23	1	1	1	1	1	1	1	1	1	1	1	1	1	1	1	1
24	1	1	1	1	1	1	1	1	1	1	1	1	1	1	1	1
25	1	1	1	1	1	1	1	1	1	1	1	1	1	1	1	1
26	1	1	1	1	1	1	1	1	1	1	1	1	1	1	1	1
27	1	1	1	1	1	1	1	1	1	1	1	1	1	1	1	1
28	1	1	1	1	1	2	1	1	1	1	1	1	1	1	1	1
29	1	1	1	1	1	1	1	1	1	1	1	1	1	1	1	1
30	1	1	1	1	1	1	1	1	1	1	1	1	1	1	1	1
31	1	1	1	1	1	1	2	2	2	1	1	1	1	1	1	1
32	1	1	1	1	1	1	1	1	1	1	1	1	1	1	1	1
33	1	1	1	1	1	1	1	1	1	1	1	1	1	1	1	1
34	1	1	1	1	1	1	1	1	1	2	1	1	1	1	1	1
35	1	1	1	1	1	1	1	1	1	1	1	1	1	1	1	1
36	1	1	1	1	1	1	1	1	1	1	1	1	1	1	1	1
37	1	1	1	1	1	1	1	1	1	1	2	1	1	1	1	1
38	1	1	1	1	1	1	1	1	1	1	1	1	1	1	1	1
39	1	1	1	1	1	1	1	1	1	1	1	1	1	1	1	1
40	1	1	1	1	1	1	1	1	1	1	1	1	1	1	1	1
41	1	1	1	1	1	1	1	1	1	1	1	1	1	1	1	1
42	1	1	1	1	1	1	1	1	1	1	1	1	1	1	1	1
43	1	1	1	1	1	1	1	1	1	1	1	1	1	1	1	1
44	2	2	1	1	1	1	1	1	1	1	1	1	1	1	1	1
45	1	1	1	1	1	1	1	1	1	1	1	1	1	1	1	1
46	1	1	1	1	1	1	1	1	1	1	1	1	1	1	1	1

47	1	1	1	1	1	1	1	1	1	1	1	1	1	1	1	1
48	1	1	1	1	1	1	1	1	1	1	1	1	1	1	1	1
49	1	1	1	1	1	1	1	1	1	1	1	1	1	1	1	1
50	1	1	1	1	1	1	1	1	1	1	1	2	1	1	1	1
51	1	1	1	1	1	1	1	1	1	1	1	1	1	1	1	1
52	1	1	1	1	1	1	1	1	1	1	1	1	1	1	1	1
53	1	1	1	1	1	1	1	1	1	1	1	1	1	1	1	1
54	1	1	1	1	1	1	1	1	1	1	1	1	1	1	1	1
55	1	1	1	1	1	1	1	1	1	1	1	1	1	1	1	1
56	1	1	1	1	1	1	1	1	1	1	1	1	1	1	1	1
57	1	1	1	1	1	1	1	1	1	1	1	1	2	1	1	1
58	1	1	1	1	1	1	1	1	1	1	1	1	1	1	1	1
59	1	1	1	1	1	1	1	1	1	1	1	1	1	1	1	1
60	1	1	1	1	1	1	1	1	1	1	1	1	1	1	1	1
61	1	1	1	1	1	1	1	1	1	1	1	1	1	2	1	1
62	1	1	1	1	1	1	1	1	1	1	1	1	1	1	1	1
63	1	1	1	1	1	1	1	1	1	1	1	1	1	1	1	1
64	1	1	1	1	1	1	1	1	1	1	1	1	1	1	2	1
65	1	1	1	1	1	1	1	1	1	1	1	1	1	1	1	1
66	1	1	1	1	1	1	1	1	2	1	1	1	1	1	1	1
67	1	1	1	1	1	1	1	1	1	1	1	1	1	1	1	1
68	1	1	1	1	1	1	1	1	1	1	1	1	1	1	1	1
69	1	1	1	1	1	1	1	1	1	1	1	1	1	1	1	1
70	1	1	1	1	1	1	1	1	1	1	1	1	1	1	1	1
71	1	1	1	1	1	1	1	1	1	1	1	1	1	1	1	1
72	1	1	1	1	1	1	1	1	1	1	1	1	1	1	1	1
73	1	1	2	1	1	1	1	1	1	1	1	1	1	1	1	2
74	1	1	1	1	1	1	1	1	1	1	1	1	1	1	1	1
75	1	1	1	1	1	1	1	1	1	1	1	1	1	1	1	1
76	1	1	1	1	1	1	1	1	1	1	1	1	1	1	1	1
77	1	1	1	1	1	1	1	1	1	1	1	1	1	1	1	1
78	1	1	1	1	1	1	1	1	1	1	1	1	1	1	1	1
79	1	1	1	2	1	1	1	1	1	1	1	1	1	1	1	1
80	1	1	1	1	1	1	1	1	1	1	1	1	1	1	1	1
81	1	1	1	1	1	1	1	1	1	1	1	1	1	1	1	1
82	1	1	1	1	1	1	1	1	1	1	1	1	1	1	1	1
83	1	1	1	1	1	1	1	1	1	1	1	1	1	1	1	1
84	1	1	1	1	1	1	1	1	1	1	1	1	1	1	1	1
85	1	1	1	1	1	1	1	1	1	1	1	1	1	1	1	1
86	1	1	1	1	1	1	1	1	1	1	1	1	1	1	2	1
87	1	1	1	1	1	1	1	1	1	1	1	1	1	1	1	1
88	1	1	1	1	1	1	1	1	1	1	1	1	1	1	1	1
89	1	1	1	1	1	1	1	1	1	1	1	1	1	1	1	1
90	1	1	1	2	1	1	1	1	1	1	1	1	1	1	1	1
91	1	1	1	1	1	1	1	1	1	1	1	1	1	1	1	1
92	1	1	1	1	1	1	1	1	1	1	1	1	1	1	1	1
93	1	1	1	1	1	1	1	1	1	2	1	1	1	1	1	1
94	1	1	1	1	1	1	1	1	1	2	1	1	1	1	1	1
95	1	1	1	1	1	1	1	1	1	1	1	1	1	1	1	1
96	1	1	1	1	1	1	1	1	1	1	1	1	1	1	1	1
97	1	1	1	1	1	1	1	1	1	1	1	1	1	1	1	1
98	1	1	1	1	1	1	1	1	1	1	1	1	1	1	1	1
99	1	1	1	1	1	1	1	1	1	1	1	1	1	1	1	1
100	1	1	1	1	1	1	1	1	1	1	1	1	1	1	1	1
101	1	1	1	1	1	1	1	1	1	1	2	1	1	1	1	1
102	1	1	1	1	1	1	1	1	1	1	1	1	1	1	1	1

103	1	1	1	1	1	1	1	1	1	1	1	1	1	1	1	1
104	1	1	1	1	1	1	1	1	1	1	1	1	1	1	1	1
105	1	1	1	1	1	1	1	1	1	1	1	1	1	1	1	1
106	1	1	1	1	1	1	1	1	1	1	1	1	1	1	1	1
107	1	1	1	1	1	1	1	1	1	1	1	1	1	1	1	1
108	1	1	1	1	1	1	1	1	1	1	1	1	1	1	1	1
109	1	1	1	1	1	1	1	1	1	1	1	1	1	1	1	1
110	1	1	1	1	1	1	1	1	1	1	1	1	1	1	1	1
111	1	1	1	1	1	1	1	1	1	1	1	1	1	1	1	1
112	1	1	1	1	1	1	1	1	1	1	1	1	1	1	1	1
113	1	1	1	1	1	1	1	1	1	1	1	1	1	1	1	1
114	1	1	1	1	1	1	1	1	1	1	1	1	1	1	1	1
115	1	1	1	1	1	1	1	1	1	1	1	1	1	1	1	1
116	1	1	1	1	1	1	1	1	1	1	1	1	1	1	1	1
117	1	1	1	1	1	1	1	1	1	1	1	1	1	1	1	1
118	1	1	1	1	1	1	1	1	1	1	1	1	1	1	1	1
119	1	1	1	1	1	1	1	1	1	1	1	1	1	1	1	1
120	1	1	1	1	1	1	1	1	1	1	1	1	1	1	1	1
121	1	1	1	1	1	1	1	1	1	1	1	1	1	1	1	1
122	1	1	1	1	1	1	1	1	1	1	1	1	1	1	1	1
123	1	1	1	1	1	1	1	1	1	1	1	1	1	1	1	1
124	1	1	1	1	1	1	1	1	1	1	1	1	1	1	1	1
125	1	1	1	1	1	1	1	1	1	1	1	1	1	1	1	1
126	1	1	1	1	1	1	1	1	1	1	1	1	1	1	1	1
127	1	1	1	1	1	1	1	1	1	1	1	1	1	1	1	1
128	1	1	1	1	1	1	1	1	1	1	1	1	1	1	1	1
129	1	1	1	1	1	1	1	1	1	1	1	1	1	1	1	1
130	1	1	1	1	1	1	1	1	1	1	1	1	1	1	1	1
131	1	1	1	1	1	1	1	1	1	1	1	1	1	1	1	1
132	1	1	1	1	1	1	1	1	1	1	1	1	1	1	1	1
133	1	1	1	1	1	1	1	1	1	1	1	1	1	1	1	1
134	1	1	1	1	1	1	1	1	1	1	1	1	1	1	1	1
135	1	1	1	1	1	1	1	1	1	1	1	1	1	1	1	1
136	1	1	1	1	1	1	1	1	1	1	1	1	1	1	1	1
137	1	1	1	1	1	1	1	1	1	1	1	1	1	1	1	1
138	1	1	1	1	1	1	1	1	1	1	1	1	1	1	1	1
139	1	1	1	1	1	1	1	1	1	1	1	1	1	1	1	1
140	1	1	1	1	1	1	1	1	1	1	1	1	1	1	1	1
141	1	1	1	1	1	1	1	1	1	1	1	1	1	1	1	1
142	1	1	1	1	1	1	1	1	1	1	1	1	1	1	1	1
143	1	1	1	1	1	1	1	1	1	1	1	1	1	1	1	1
144	1	1	1	1	1	1	1	1	1	1	1	1	1	1	1	1
145	1	1	1	1	1	1	1	1	1	1	1	1	1	1	1	1
146	1	1	1	1	1	1	1	1	1	1	1	1	1	1	1	1
147	1	1	1	1	1	1	1	1	1	1	1	1	1	1	1	1
148	1	1	1	1	1	1	1	1	1	1	1	1	1	1	1	1
149	1	1	1	1	1	1	1	1	1	1	1	1	1	1	1	1
150	1	1	1	1	1	1	1	1	1	1	1	1	1	1	1	1
151	1	1	1	1	1	1	1	1	1	1	1	1	1	2	1	1
152	1	1	1	1	1	1	1	1	2	1	1	1	1	1	1	1
153	1	1	1	1	1	1	1	1	1	1	1	1	1	1	1	1
154	1	1	1	1	1	1	1	1	1	1	1	1	1	1	1	1
155	1	1	1	1	1	1	1	1	1	1	1	1	1	1	1	1
156	1	1	1	1	1	1	1	1	1	1	1	1	1	1	1	1
157	1	1	1	1	1	1	1	1	1	1	1	1	1	1	1	1
158	1	1	1	1	1	1	1	1	1	1	1	1	1	1	1	1

159	1	1	1	1	1	1	1	1	1	1	1	1	1	1	1	1
160	1	1	1	1	1	1	1	1	1	1	1	1	1	1	1	2
161	1	1	1	1	1	1	1	1	1	1	1	1	1	1	1	1
162	1	1	1	1	1	1	1	1	1	1	1	1	1	1	1	1
163	1	1	1	1	1	1	1	1	1	1	1	1	1	1	1	1
164	1	1	1	1	1	1	1	1	1	1	1	1	1	1	1	1
165	1	1	1	1	1	1	1	1	1	1	1	1	1	1	1	1
166	1	1	1	1	1	1	1	1	1	1	1	1	1	1	1	1
167	1	1	1	1	1	1	1	1	1	1	1	1	1	1	1	1
168	1	1	1	1	1	1	1	1	1	1	1	1	1	1	1	1
169	1	1	1	1	1	1	1	1	1	1	1	1	1	1	1	1
170	1	1	1	1	1	1	1	1	1	1	1	1	1	1	1	1
171	1	1	1	1	1	1	1	1	1	1	1	1	1	1	1	1
172	1	1	1	1	1	1	1	1	1	1	1	1	1	1	1	1
173	1	1	1	1	1	1	1	1	1	1	1	1	1	1	1	1
174	1	1	1	1	1	1	1	1	1	1	1	1	1	1	1	1
175	1	1	1	1	1	1	1	1	2	1	1	1	1	1	1	1
176	1	1	1	1	1	1	1	1	1	1	1	1	1	1	1	1
177	1	1	1	1	1	1	1	1	1	1	1	1	1	1	1	1
178	1	1	1	1	1	1	1	1	1	1	1	1	1	1	1	1
179	1	1	1	1	1	1	1	1	1	1	1	1	1	1	1	1
180	1	1	1	1	1	1	1	1	1	1	1	1	1	1	1	1
181	1	1	1	1	1	1	1	1	1	1	1	1	1	1	1	1
182	1	1	1	1	1	1	1	1	1	1	1	1	1	1	1	1
183	1	1	1	1	1	1	1	1	1	1	1	1	1	1	1	1
184	1	1	1	1	1	1	1	1	1	1	1	1	1	1	1	1
185	1	1	1	1	1	1	1	1	1	1	1	1	1	1	1	1
186	1	1	1	1	1	1	1	1	1	1	1	1	1	1	1	1
187	1	1	1	1	1	1	1	1	1	1	1	1	1	1	1	1
188	1	1	1	1	1	1	1	1	1	1	1	1	1	1	1	1
189	1	1	1	1	1	1	1	1	1	1	1	1	1	1	1	1
190	1	1	1	1	1	1	1	1	1	1	1	1	1	1	1	1
191	1	1	1	1	1	1	1	1	1	1	1	1	1	1	1	1
192	1	1	1	1	1	1	1	1	1	1	1	1	1	1	1	1
193	1	1	1	1	1	1	1	1	1	1	1	1	1	1	1	1
194	1	1	1	1	1	1	1	1	1	1	1	1	1	1	1	1
195	1	1	1	1	1	1	1	1	1	1	1	1	1	1	1	1
196	1	1	1	1	1	1	1	1	1	1	1	1	1	1	1	1
197	2	1	1	1	1	1	1	1	1	1	1	1	1	1	1	1
198	1	1	1	1	1	1	1	1	1	1	1	1	1	1	1	1
199	1	1	1	1	1	1	1	1	1	1	1	1	1	1	1	1
200	1	1	1	1	1	1	1	1	1	1	1	1	1	1	1	1
201	1	1	1	1	1	1	1	1	1	1	1	1	1	1	1	1
202	1	1	1	1	1	1	1	1	1	1	1	1	1	2	1	1
203	1	1	1	1	1	1	1	1	1	1	1	1	1	1	1	1
204	1	1	1	1	1	1	1	1	1	1	1	1	1	1	1	1
205	1	1	1	1	1	1	1	1	1	1	1	1	1	1	1	1
206	1	1	1	1	1	1	1	1	1	1	1	1	1	1	1	1
207	1	1	1	1	1	1	1	1	1	1	1	1	1	1	1	1
208	1	1	1	1	1	1	1	1	1	1	1	1	1	1	1	1
209	1	1	1	1	1	1	1	1	1	1	1	1	1	1	1	1
210	1	1	1	1	1	1	1	1	1	1	1	1	1	1	1	1
211	1	1	1	1	1	1	1	1	1	1	1	1	1	1	1	1
212	1	1	1	1	1	1	1	1	1	1	1	1	1	2	1	1
213	1	1	1	1	1	1	1	1	1	1	1	1	1	1	1	1
214	1	1	1	1	1	1	1	1	1	1	1	1	1	1	1	1

215	1	1	1	1	1	1	1	1	1	1	1	1	1	1	1	1
216	1	1	1	1	1	1	1	1	1	1	1	1	1	1	1	1
217	1	1	1	1	1	1	1	1	1	1	1	1	1	1	1	1
218	1	1	1	1	1	1	1	1	1	1	1	1	1	1	1	1
219	1	1	1	1	1	1	1	1	1	1	1	1	1	1	1	1
220	1	1	1	1	1	1	1	1	1	1	1	1	1	1	1	1
221	1	1	1	1	1	1	1	1	1	1	1	1	1	1	1	1
222	1	1	1	1	1	1	1	1	1	1	1	1	1	1	1	1
223	1	1	1	1	1	1	1	1	1	1	1	1	1	1	1	1
224	1	1	1	1	1	1	1	1	1	1	1	1	1	1	1	1
225	1	1	1	1	1	1	1	1	1	1	1	1	1	1	1	1
226	1	1	1	1	1	1	1	1	1	1	1	1	1	1	1	1
227	1	1	1	1	1	1	1	1	1	1	1	1	1	1	1	1
228	1	1	1	1	1	1	1	1	1	1	1	1	1	1	1	1
229	1	1	1	1	1	1	1	1	1	1	1	1	1	1	1	1
230	1	1	1	1	1	1	1	1	1	1	1	1	1	1	1	1
231	1	1	1	1	1	1	1	1	1	1	1	1	1	1	1	1
232	1	1	1	1	1	1	1	1	1	1	1	1	1	1	1	1
233	1	1	1	1	1	1	1	1	1	1	1	1	1	1	1	1
234	1	1	1	1	1	1	1	1	1	1	1	1	1	1	1	1
235	1	1	1	1	1	1	1	1	1	1	1	1	1	1	1	1
236	1	1	1	1	1	1	1	1	1	1	1	1	1	1	1	1
237	1	1	1	1	1	1	1	1	1	1	1	1	1	1	1	1
238	1	1	1	1	1	1	1	1	1	1	1	1	1	1	1	1
239	1	1	1	1	1	1	1	1	1	1	1	1	1	1	1	1
240	1	1	1	1	1	1	1	1	1	1	1	1	1	1	1	1
241	1	1	1	1	1	1	1	1	1	1	1	1	1	1	1	1
242	1	1	1	1	1	1	1	1	1	1	1	1	1	1	1	1
243	1	1	1	1	1	1	1	1	1	1	1	1	1	1	1	1
244	1	1	1	1	1	1	1	1	1	1	1	1	1	1	1	1
245	1	1	1	1	1	1	1	1	1	1	1	1	1	1	1	1
246	1	1	1	1	1	1	1	1	1	1	1	1	1	1	1	1
247	1	1	1	1	1	1	1	1	1	1	1	1	1	1	1	1
248	1	1	1	1	1	1	1	1	1	1	1	1	1	1	1	1
249	1	1	1	1	1	1	1	1	1	1	1	1	1	1	1	1
250	1	1	1	1	1	1	1	1	1	1	1	1	1	1	1	1
251	1	1	1	1	1	1	1	1	1	1	1	1	1	1	1	1
252	1	1	1	1	1	1	1	1	1	1	1	1	1	1	1	1
253	1	1	1	1	1	1	1	1	1	1	1	1	1	1	1	1
254	1	1	1	1	1	1	1	1	1	1	1	1	1	1	1	1
255	1	1	1	1	1	1	1	1	1	1	1	1	1	1	1	1
256	1	1	1	1	1	2	1	1	1	1	1	1	1	1	1	1
257	1	1	1	1	1	1	1	1	1	1	1	1	1	1	1	1
258	1	1	1	1	1	1	1	1	1	1	1	1	1	1	1	1
259	1	1	1	1	1	1	1	1	1	1	1	1	1	1	1	1
260	1	1	1	1	1	1	1	1	1	1	1	1	1	1	1	1
261	1	1	1	1	1	1	1	1	1	1	1	1	1	1	1	1
262	1	1	1	1	1	1	1	1	1	1	1	1	1	1	1	1
263	1	1	1	1	1	1	1	1	1	1	1	1	1	1	1	1
264	1	1	1	1	1	1	1	1	1	1	1	1	1	1	1	1
265	1	1	1	1	1	1	1	1	1	1	1	1	1	1	1	1
266	1	1	1	1	1	1	1	1	1	1	1	1	1	1	1	1
267	1	1	1	1	1	1	1	1	1	1	1	1	1	1	1	1
268	1	1	2	1	1	1	1	1	1	1	1	1	1	1	1	1

ID: Identity number, CAZ: Ceftazidime, A.S.A: Aspirin

EXCLI Journal 2021;20:444-453 – ISSN 1611-2156
Received: January 16, 2021, accepted: February 16, 2021, published: February 22, 2021

Supplementary Table 8: Raw data of in-hospital management of SARS-CoV-2 infected patients with or without diabetes which is shown in Table 2 of the manuscript (part 4).

ID	Atenolol 1: No 2: Yes	Omepra-zole 1: No 2: Yes	Fa-motidine 1: No 2: Yes	Inderal 1: No 2: Yes	Salbuta-mol 1: No 2: Yes	Ribavi-rin 1: No 2: Yes	oseltami-vir 1: No 2: Yes	Cipro 1: No 2: Yes	Kaletra 1: No 2: Yes	IV 1: No 2: Yes
1	1	1	1	1	1	1	1	1	1	1
2	1	1	1	1	1	1	1	1	2	1
3	1	1	1	1	1	1	1	1	2	2
4	1	1	1	1	1	1	1	1	2	1
5	1	1	1	1	1	1	1	1	1	1
6	1	1	1	1	1	1	1	1	1	1
7	1	1	1	1	1	1	1	1	1	1
8	1	1	1	1	1	1	1	1	1	1
9	1	1	1	1	1	1	1	1	2	1
10	1	1	1	1	1	1	1	1	1	1
11	1	1	1	1	1	1	1	1	1	1
12	1	1	1	1	1	1	1	1	1	1
13	1	1	1	1	1	1	1	1	2	1
14	1	1	1	1	1	1	1	1	1	1
15	1	1	1	1	1	1	1	1	2	1
16	1	1	1	1	1	1	1	1	1	1
17	1	1	1	1	1	1	1	1	1	1
18	1	1	1	1	1	1	1	1	1	1
19	1	1	1	1	1	1	1	1	2	1
20	1	1	1	1	1	1	1	1	1	1
21	1	1	1	1	1	1	1	1	2	1
22	1	1	1	1	1	1	1	1	2	1
23	1	1	1	1	1	1	1	1	2	1
24	1	1	1	1	1	1	1	1	1	1
25	1	1	1	1	1	1	1	1	2	1
26	1	1	1	1	1	1	1	1	2	1
27	1	1	1	1	1	1	1	1	1	1
28	1	1	1	1	1	1	1	1	1	2
29	1	1	1	1	1	1	1	1	1	1
30	1	1	1	1	1	1	1	1	1	1
31	1	1	1	1	1	1	1	1	2	1
32	1	1	1	1	1	1	1	1	2	1
33	1	1	1	1	1	1	1	1	2	1
34	1	1	1	1	1	1	1	1	1	1
35	1	1	1	1	1	1	1	1	1	1
36	1	1	1	1	1	1	1	1	2	1
37	1	1	1	1	1	1	1	1	1	1
38	1	1	1	1	1	1	1	1	1	1
39	1	1	1	1	1	1	1	1	1	1
40	1	1	1	1	1	1	1	1	1	1
41	1	1	1	1	1	1	1	1	1	1
42	1	1	1	1	1	1	1	1	1	1
43	1	1	1	1	1	1	1	1	1	1
44	1	1	1	1	1	1	1	1	2	1
45	1	1	1	1	1	1	1	1	1	1
46	1	1	1	1	1	1	1	1	1	1
47	1	1	1	1	1	1	1	1	1	1
48	1	1	1	1	1	1	1	1	2	1
49	1	1	1	1	1	1	1	1	2	1
50	1	1	1	1	1	1	1	1	1	1
51	1	1	1	1	1	1	1	1	1	1
52	1	1	1	1	1	1	1	1	1	1
53	1	1	1	1	1	1	1	1	1	1
54	1	1	1	1	1	1	1	1	2	1
55	1	1	1	1	1	1	1	1	1	1
56	1	1	1	1	1	1	1	1	1	1
57	1	1	1	1	1	1	1	1	1	1
58	1	1	1	1	1	1	1	1	1	1
59	1	1	1	1	1	1	1	1	1	1
60	1	1	1	1	1	1	1	1	2	1
61	1	1	1	1	1	1	1	1	1	1
62	1	1	1	1	1	1	1	1	1	1
63	1	1	1	1	1	1	1	1	1	1
64	1	1	1	1	1	1	1	1	1	1
65	1	1	1	1	1	1	1	1	1	1
66	1	1	1	1	1	1	1	1	1	1
67	1	1	1	1	1	1	1	1	1	1
68	1	1	1	1	1	1	1	1	2	1
69	1	1	1	1	1	1	1	1	2	1
70	1	1	1	1	1	1	1	1	1	1
71	1	1	1	1	1	1	1	1	1	1
72	1	1	1	1	1	1	1	1	1	1
73	2	1	1	1	1	1	1	1	1	1
74	1	1	1	1	1	1	1	1	1	1
75	1	1	1	1	1	1	1	1	1	1
76	1	1	1	1	1	1	1	1	1	1

EXCLI Journal 2021;20:444-453 – ISSN 1611-2156
Received: January 16, 2021, accepted: February 16, 2021, published: February 22, 2021

77	1	1	1	1	1	1	1	1	1	1
78	1	1	1	1	1	1	1	1	1	1
79	1	1	1	1	1	1	1	1	1	1
80	1	1	1	1	1	1	1	2	1	1
81	1	1	1	1	1	1	1	1	2	1
82	1	1	1	1	1	1	1	1	2	1
83	1	1	1	1	1	1	1	1	2	1
84	1	1	1	1	1	1	1	1	2	1
85	1	1	1	1	1	1	1	1	1	1
86	1	1	1	1	1	1	1	1	2	1
87	1	1	1	1	1	1	1	1	1	1
88	1	1	1	1	1	1	1	1	1	1
89	1	2	1	1	1	1	1	1	1	1
90	1	1	1	1	1	1	1	1	1	1
91	1	1	1	1	1	1	1	1	2	1
92	1	1	1	1	1	1	1	1	1	1
93	1	1	1	1	1	1	1	1	1	1
94	1	1	1	1	1	1	1	1	2	1
95	1	1	2	1	1	1	1	1	2	1
96	1	1	1	1	1	1	1	1	1	1
97	1	1	2	2	1	1	1	1		1
98	1	1	1	1	1	1	1	1	1	1
99	1	1	1	1	1	1	1	1	1	1
100	1	1	1	1	1	1	1	1	2	1
101	1	1	1	1	1	1	1	1	1	1
102	1	1	1	1	1	1	1	1	2	1
103	1	1	1	1	1	1	1	1	1	1
104	1	1	1	1	1	1	1	1	1	1
105	1	1	1	1	1	1	1	1	2	1
106	1	1	1	1	1	1	1	1	2	1
107	1	1	1	1	1	1	1	1	1	1
108	1	1	1	1	1	1	1	1	1	1
109	1	1	1	1	1	1	1	1	1	1
110	1	1	1	1	1	1	1	1	1	1
111	1	1	1	1	1	1	1	1	1	1
112	1	1	1	1	1	1	1	1	2	1
113	1	1	1	1	1	1	1	1	1	2
114	1	1	1	1	1	1	1	1	1	2
115	1	1	1	1	1	1	1	1	1	1
116	1	1	1	1	1	1	1	1	2	1
117	1	1	1	1	1	1	1	1	2	1
118	1	1	1	1	1	1	1	1	1	1
119	1	1	1	1	1	1	1	1	1	1
120	1	1	1	1	1	1	1	1	1	1
121	1	1	1	1	1	1	1	1	1	1
122	1	1	1	1	1	1	1	1	1	1
123	1	1	1	1	1	1	1	1	1	1
124	1	1	1	1	1	1	1	1	1	1
125	1	1	1	1	1	1	1	1	1	1
126	1	1	1	1	1	1	1	1	2	1
127	1	1	1	1	1	1	1	1	1	1
128	1	1	1	1	1	1	1	1	2	1
129	1	1	1	1	1	1	1	1	1	1
130	1	1	1	1	1	1	1	1	2	1
131	1	1	1	1	1	1	1	1	1	1
132	1	1	1	1	1	1	1	1	1	1
133	1	1	1	1	1	1	1	1	2	1
134	1	1	1	1	1	1	1	1	1	1
135	1	1	1	1	1	1	1	1	1	1
136	1	1	1	1	1	1	1	1	2	1
137	1	1	1	1	1	1	1	1	1	1
138	1	1	1	1	1	1	1	1	1	1
139	1	1	1	1	1	1	1	1	2	1
140	1	1	1	1	1	1	1	1	1	1
141	1	1	1	1	1	1	1	1	1	1
142	1	1	1	1	1	1	1	1	2	1
143	1	1	1	1	1	1	1	1	1	1
144	1	1	1	1	1	1	1	1	2	1
145	1	1	1	1	1	1	1	1	2	1
146	1	1	1	1	1	1	1	1	1	1
147	1	1	1	1	1	1	1	1	2	1
148	1	1	1	1	1	1	1	1	2	1
149	1	1	1	1	1	1	1	1	1	1
150	1	1	1	1	1	1	1	1	2	1
151	1	1	1	1	1	1	1	1	2	1
152	1	1	1	1	1	1	1	1	2	1
153	1	1	1	1	1	1	1	1	1	1
154	1	1	1	1	1	1	1	1	2	1
155	1	1	1	1	1	1	1	1	2	1
156	1	1	1	1	1	1	1	1	2	1
157	1	1	1	1	1	1	1	1	2	1
158	1	1	1	1	1	1	1	1	1	1
159	1	1	1	1	1	1	1	1	1	1
160	1	1	1	1	1	1	1	1	2	1
161	1	1	1	1	1	1	1	1	2	2

EXCLI Journal 2021;20:444-453 – ISSN 1611-2156
Received: January 16, 2021, accepted: February 16, 2021, published: February 22, 2021

162	1	1	1	1	1	1	1	1	1	1
163	1	1	1	1	1	1	1	1	2	1
164	1	1	1	1	1	1	1	1	2	2
165	1	1	1	1	1	1	1	1	1	1
166	1	1	1	1	1	1	1	1	1	1
167	1	1	1	1	1	1	1	1	2	1
168	1	1	1	1	1	1	1	1	1	1
169	1	1	1	1	1	1	1	1	1	1
170	1	1	1	1	1	1	1	1	2	1
171	1	1	1	1	1	1	1	1	2	1
172	1	1	1	1	1	1	1	1	2	1
173	1	1	1	1	1	1	1	1	2	1
174	1	1	1	1	1	1	1	1	1	1
175	1	1	1	1	1	1	1	1	2	1
176	1	1	1	1	1	1	1	1	2	1
177	1	1	1	1	1	1	1	1	2	1
178	1	1	1	1	1	1	1	1	2	1
179	1	1	1	1	1	1	1	1	2	1
180	1	1	1	1	1	1	1	1	1	1
181	1	1	1	1	1	1	1	1	1	1
182	1	1	1	1	1	1	1	1	2	1
183	1	1	1	1	1	1	1	1	2	1
184	1	1	1	1	1	1	1	1	2	2
185	1	1	1	1	1	1	1	1	1	1
186	1	1	1	1	1	1	1	1	2	1
187	1	1	1	1	1	1	1	1	1	1
188	1	1	1	1	1	1	1	1	1	1
189	1	1	1	1	1	1	1	1	2	2
190	1	1	1	1	1	1	1	1	2	1
191	1	1	1	1	1	1	1	1	2	1
192	1	1	1	1	1	1	1	1	2	1
193	1	1	1	1	1	1	1	1	2	1
194	1	1	1	1	1	1	1	1	1	1
195	1	1	1	1	1	1	1	1	2	2
196	1	1	1	1	1	1	1	1	2	1
197	1	1	1	1	2	1	1	1	2	1
198	1	1	1	1	1	1	1	1	2	1
199	1	1	1	1	1	1	1	1	2	1
200	1	1	1	1	1	1	1	1	1	2
201	1	1	1	1	1	1	1	1	1	1
202	1	1	1	1	1	1	1	1	1	1
203	1	1	1	1	1	1	1	1	2	1
204	1	1	1	1	1	1	1	1	2	1
205	1	1	1	1	1	1	1	1	2	1
206	1	1	1	1	1	1	1	1	1	2
207	1	1	1	1	1	2	2	1	2	2
208	1	1	1	1	1	1	2	1	2	1
209	1	1	1	1	1	1	1	1	1	1
210	1	1	1	1	1	1	1	1	1	1
211	1	1	1	1	1	1	1	1	2	1
212	1	1	1	1	1	1	1	1	2	1
213	1	1	1	1	1	1	1	1	2	1
214	1	1	1	1	1	1	1	1	2	1
215	1	1	1	1	1	1	1	1	1	1
216	1	1	1	1	1	1	1	1	2	1
217	1	1	1	1	1	1	1	1	2	1
218	1	1	1	1	1	1	1	1	2	1
219	1	1	1	1	1	1	1	1	2	1
220	1	1	1	1	1	1	1	1	2	1
221	1	1	1	1	2	1	1	1	2	2
222	1	1	1	1	1	1	1	1	2	1
223	1	1	1	1	1	1	1	1	2	1
224	1	1	1	1	1	1	1	1	2	1
225	1	1	1	1	1	1	1	1	2	1
226	1	1	1	1	1	1	1	1	2	1
227	1	1	1	1	1	1	1	1	2	1
228	1	1	1	1	1	1	1	1	2	2
229	1	1	1	1	1	1	1	1	2	2
230	1	1	1	1	1	1	1	1	1	1
231	1	1	1	1	1	1	1	1	2	2
232	1	1	1	1	1	1	1	1	2	2
233	1	1	1	1	1	1	1	1	2	1
234	1	1	1	1	1	1	1	1	2	1
235	1	1	1	1	1	1	1	1	2	1
236	1	1	1	1	1	1	1	1	2	1
237	1	1	1	1	1	1	1	1	2	1
238	1	1	1	1	1	1	1	1	1	1
239	1	1	1	1	1	1	2	1	2	1
240	1	1	1	1	1	1	1	1	2	1
241	1	1	1	1	1	1	1	1	1	1
242	1	1	1	1	1	1	1	1	1	1
243	1	1	1	1	1	1	1	1	1	1
244	1	1	1	1	1	1	1	1	1	1
245	1	1	1	1	1	1	1	1	2	1
246	1	1	1	1	1	1	1	1	1	1

EXCLI Journal 2021;20:444-453 – ISSN 1611-2156
Received: January 16, 2021, accepted: February 16, 2021, published: February 22, 2021

247	1	1	1	1	1	1	1	1	1	1
248	1	1	1	1	1	1	1	1	2	2
249	1	1	1	1	1	2	1	1	1	2
250	1	1	1	1	1	1	1	1	2	2
251	1	1	1	1	1	1	1	1	1	2
252	1	1	1	1	1	2	2	1	1	2
253	1	1	1	1	1	1	1	1	2	2
254	1	1	1	1	1	1	1	1	2	2
255	1	1	1	1	1	1	1	1	1	2
256	1	1	1	1	1	1	1	1	2	2
257	1	1	1	1	1	1	1	1	1	2
258	1	1	1	1	1	1	1	1	2	1
259	1	1	1	1	1	1	1	1	2	1
260	1	1	1	1	1	1	1	1	2	1
261	1	1	1	1	1	1	1	1	2	1
262	1	1	1	1	1	1	1	1	2	1
263	1	1	1	1	1	1	2	1	2	1
264	1	1	1	1	1	1	1	1	2	1
265	1	1	1	1	1	1	1	1	1	1
266	1	1	1	1	1	1	1	1	2	1
267	1	1	1	1	1	1	1	1	1	1
268	1	1	1	1	1	1	1	1	2	1

ID: Identity number, Cipro: Ciprofloxacin, IV: Invasive ventilation

EXCLI Journal 2021;20:444-453 – ISSN 1611-2156
Received: January 16, 2021, accepted: February 16, 2021, published: February 22, 2021

Supplementary Table 9: Raw data of multivariable analyses between patients with and without diabetes which is shown in Table 3 of the manuscript.

ID	LOS	IMV<br>1: No<br>2: Yes	HM<br>1: Death<br>2: Discharged
1	5	1	2
2	3	1	2
3	5	2	2
4	3	1	2
5	3	1	2
6	7	1	2
7	7	1	1
8	8	1	2
9	5	1	2
10	1	1	2
11	5	1	2
12	4	1	2
13	4	1	2
14	4	1	1
15	4	1	2
16	6	1	2
17	9	1	2
18	5	1	2
19	6	1	2
20	8	1	2
21	6	1	2
22	4	1	2
23	5	1	2
24	5	1	2
25	7	1	2
26	5	1	2
27	4	1	2
28	17	2	1
29	6	1	2
30	6	1	2
31	8	1	2
32	3	1	2
33	9	1	2
34	5	1	2
35	4	1	2
36	5	1	2
37	5	1	2
38	4	1	2
39	6	1	2
40	6	1	2
41	5	1	2
42	6	1	2
43	5	1	2
44	1	1	2
45	3	1	2
46	4	1	2
47	4	1	2
48	5	1	2
49	4	1	2
50	6	1	2
51	5	1	2
52	12	1	2
53	7	1	2
54	5	1	2
55	5	1	2
56	4	1	2
57	4	1	2
58	8	1	2
59	8	1	2
60	5	1	2
61	18	1	2
62	10	1	2
63	6	1	2
64	5	1	2
65	8	1	2
66	7	1	2
67	4	1	2
68	10	1	2
69	6	1	2
70	6	1	2
71	miss	1	1
72	10	1	2
73	11	1	2
74	4	1	2
75	3	1	2
76	4	1	2

EXCLI Journal 2021;20:444-453 – ISSN 1611-2156
Received: January 16, 2021, accepted: February 16, 2021, published: February 22, 2021

77	6	1	2
78	8	1	2
79	5	1	2
80	6	1	2
81	7	1	2
82	6	1	2
83	9	1	2
84	5	1	2
85	5	1	2
86	7	1	2
87	4	1	2
88	4	1	2
89	7	1	2
90	6	1	2
91	1	1	2
92	4	1	2
93	5	1	2
94	2	1	2
95	3	1	2
96	5	1	2
97	6	1	2
98	5	1	2
99	1	1	2
100	5	1	2
101	5	1	2
102	9	1	2
103	4	1	2
104	9	1	2
105	7	1	2
106	6	1	2
107	5	1	2
108	5	1	2
109	4	1	2
110	6	1	2
111	4	1	2
112	4	1	1
113	2	2	1
114	5	2	1
115	12	1	2
116	10	1	1
117	6	1	2
118	10	1	2
119	4	1	2
120	7	1	2
121	6	1	2
122	7	1	2
123	6	1	2
124	5	1	2
125	3	1	2
126	7	1	2
127	5	1	2
128	9	1	2
129	6	1	2
130	8	1	2
131	6	1	2
132	5	1	2
133	5	1	2
134	5	1	2
135	4	1	2
136	5	1	2
137	6	1	2
138	4	1	2
139	9	1	2
140	7	1	2
141	6	1	2
142	4	1	2
143	15	1	2
144	7	1	2
145	1	1	2
146	6	1	2
147	8	1	1
148	12	1	1
149	7	1	1
150	6	1	2
151	15	1	2
152	8	1	1
153	4	1	2
154	7	1	2
155	6	1	2
156	4	1	2
157	4	1	2
158	6	1	2
159	7	1	2
160	8	1	2
161	2	2	2

EXCLI Journal 2021;20:444-453 – ISSN 1611-2156
Received: January 16, 2021, accepted: February 16, 2021, published: February 22, 2021

162	6	1	2
163	3	1	2
164	4	2	2
165	5	1	2
166	7	1	2
167	3	1	2
168	5	1	2
169	6	1	2
170	12	1	2
171	16	1	2
172	2	1	2
173	11	1	2
174	20	1	2
175	12	1	2
176	5	1	2
177	3	1	2
178	1	1	2
179	5	1	2
180	6	1	2
181	2	1	2
182	2	1	2
183	4	1	2
184	4	2	1
185	4	1	2
186	6	1	2
187	8	1	2
188	4	1	2
189	4	2	2
190	5	1	2
191	1	1	2
192	7	1	2
193	6	1	2
194	6	1	2
195	8	2	2
196	13	1	2
197	6	1	2
198	5	1	2
199	6	1	2
200	8	2	1
201	13	1	2
202	10	1	2
203	7	1	2
204	7	1	2
205	8	1	2
206	3	2	1
207	3	2	1
208	8	1	2
209	12	1	2
210	9	1	2
211	9	1	2
212	20	1	2
213	10	1	2
214	8	1	2
215	8	1	2
216	14	1	2
217	21	1	2
218	10	1	2
219	9	1	2
220	13	1	2
221	13	2	2
222	11	1	2
223	7	1	2
224	7	1	2
225	3	1	2
226	12	1	2
227	19	1	2
228	4	2	1
229	6	2	1
230	5	1	2
231	2	2	1
232	1	2	1
233	5	1	2
234	5	1	2
235	2	1	2
236	9	1	2
237	8	1	2
238	5	1	2
239	5	1	2
240	7	1	2
241	4	1	2
242	4	1	2
243	4	1	2
244	14	1	2
245	11	1	2
246	5	1	2

EXCLI Journal 2021;20:444-453 – ISSN 1611-2156
Received: January 16, 2021, accepted: February 16, 2021, published: February 22, 2021

247	18	1	2
248	3	2	1
249	1	2	1
250	5	2	1
251	5	2	1
252	2	2	1
253	1	2	1
254	18	2	1
255	2	2	1
256	6	2	1
257	2	2	1
258	6	1	2
259	3	1	2
260	13	1	2
261	14	1	2
262	14	1	2
263	14	1	2
264	14	1	2
265	14	1	2
266	12	1	2
267	14	1	2
268	15	1	2

ID: Identity number, LOS: Length of hospital Stay, IMV: Invasive Mechanical Ventilation, HM: Hospital mortality
